# *Buronius manfredschmidi*—A new small hominid from the early late Miocene of Hammerschmiede (Bavaria, Germany)

**DOI:** 10.1371/journal.pone.0301002

**Published:** 2024-06-07

**Authors:** M. Böhme, D. R. Begun, A. C. Holmes, T. Lechner, G. Ferreira

**Affiliations:** 1 Department of Geosciences, Section Terrestrial Palaeoclimatology, Eberhard-Karls-Universität Tübingen, Tübingen, Germany; 2 Section Palaeontology, Senckenberg Centre for Human Evolution and Palaeoenvironment, Tübingen, Germany; 3 Department of Anthropology, University of Toronto, Toronto, Ontario, Canada; University of Haifa, Zinman Institute of Archaeology, ISRAEL

## Abstract

The known diversity of European middle and late Miocene hominids has increased significantly during the last decades. Most of these great apes were frugivores in the broadest sense, ranging from soft fruit frugivores most like chimpanzees to hard/tough object feeders like orangutans, varying in size from larger than siamangs (over 17 kg) to larger than most chimpanzees (~60–70 kg). In contrast to the frequent sympatry of hominoids in the early-to-middle Miocene of Africa, in no European Miocene locality more than one hominid taxon has been identified. Here we describe the first case of hominid sympatry in Europe from the 11.62 Ma old Hammerschmiede HAM 5 level, best known from its excellent record of *Danuvius guggenmosi*. The new fossils are consistent in size with larger pliopithecoids but differ morphologically from any pliopithecoid and from *Danuvius*. They are also distinguished from early and middle Miocene apes, share affinities with late Miocene apes, and represent a small hitherto unknown late Miocene ape *Buronius manfredschmidi*. With an estimated body mass of about 10 kg it represents the smallest known hominid taxon. The relative enamel thickness of *Buronius* is thin and contrasts with *Danuvius*, whose enamel is twice as thick. The differences between *Buronius* and *Danuvius* in tooth and patellar morphology, enamel thickness and body mass are indicative of differing adaptations in each, permitting resource partitioning, in which *Buronius* was a more folivorous climber.

## Introduction

Miocene hominoid localities become increasingly common in Europe from the late middle Miocene onwards, shortly after they become rare in Africa. Despite their frequency, richness, and in three cases an exceptional abundance of well-preserved hominoid fossils (Can Llobateres, Hammerschmiede and Rudabánya), no European locality has yielded more than one hominoid taxon. In a few cases the hominoid from a site is accompanied by a pliopithecoid, though in only one case, Rudabánya, are the two catarrhines found co-mingled in the same stratigraphic level [1, 2]. In contrast, all comparably rich early and middle Miocene hominoid sites in Africa contain at least two catarrhines and often more [3, 4].

The Hammerschmiede fossil site is best known as the *Danuvius* locality. It preserves multiple individuals of *Danuvius guggenmosi* including well-preserved postcranial bones [5]. The *Danuvius* fossils come from the 11.62 Ma old HAM 5 level at Hammerschmiede [6], which is highly constrained stratigraphically and taphonomically. The HAM 5 level contains a huge taxonomic diversity, from plants to molluscs to mammals, including many partial skeletons and well-preserved crania. From this level alone, 112 vertebrate species are known (147 from all levels together), including 73 species of mammals (84 mammals in total from the outcrop). So far, only a fraction of the enormous vertebrate fauna has been studied in detail, including carnivores [7–10], artiodactyles [11–13], beavers [14], small mammals [15–20] and birds [21–23].

HAM 5 also yielded two primate teeth and one patella that are too small and morphologically different to be attributed to *Danuvius guggenmosi* or any known European Miocene catarrhine. These specimens represent a small hitherto unknown European late Miocene ape, which we describe in this contribution. The presence of a second hominoid at HAM 5 is more consistent with the level of primate diversity found at many early Miocene localities and hence we discuss the new taxon in the light of sympatry in fossil apes.

### Geologic and taphonomic setting

**Fig 1:**
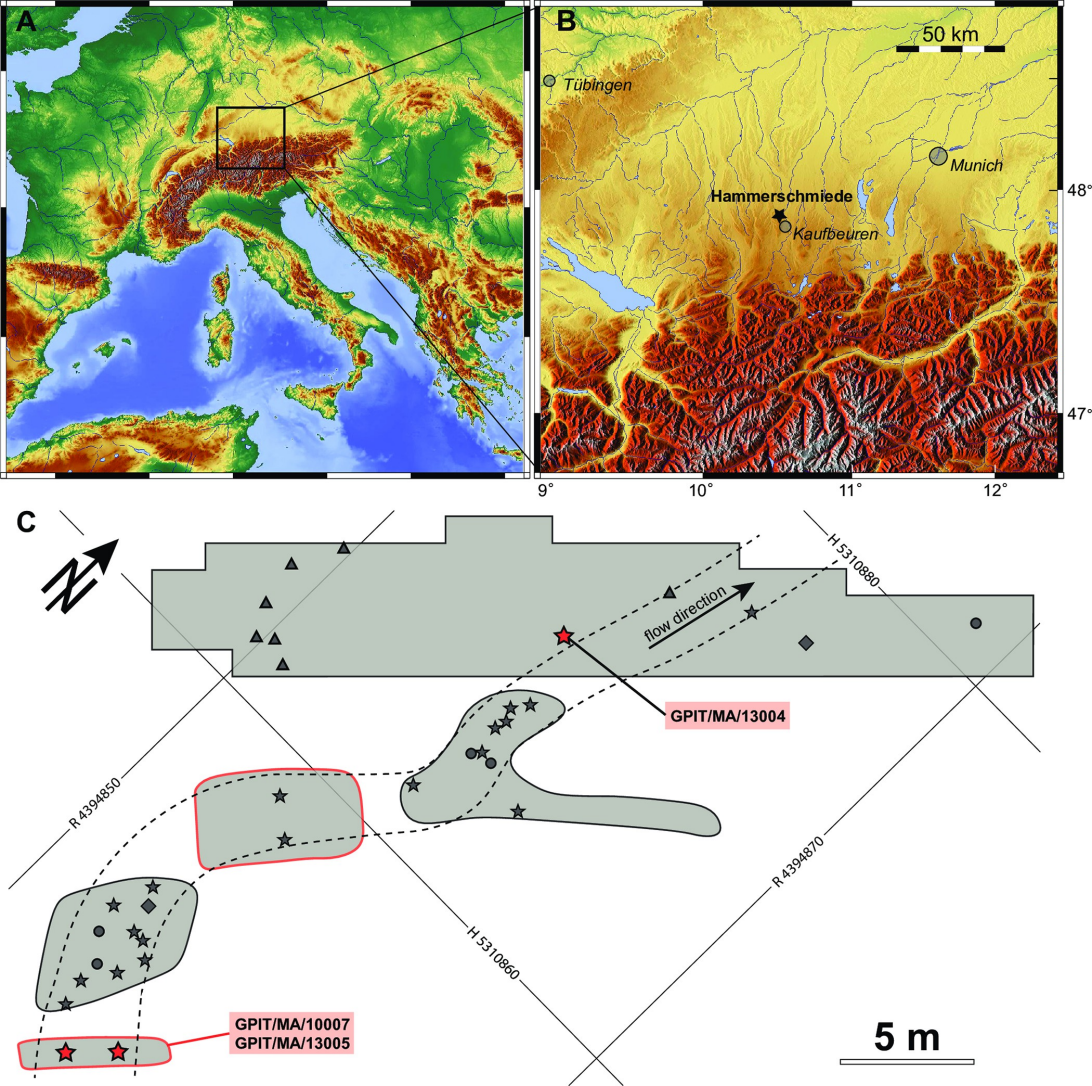
Geographical position of the Hammerschmiede locality (A, B) and excavation plan (C) of the channel structure HAM 5 (grey areas excavated from 2011 to 2019). Dashed line represents the channel structure. Red stars represent the specimens of *Buronius manfredschmidi* and grey symbols represent *Danuvius* individuals; stars–GPIT/MA/10000 (male holotype), diamonds–GPIT/MA/10001 (female paratype), circles–GPIT/MA/10002 (juvenile paratype), triangles–GPIT/MA/10003 (female paratype). Red encircled areas have no tachymeter measurements. Coordinates correspond to Gauss-Krüger Zone 4 grid with easting (R) and northing (H) in metres. The topographic maps have been created using the Generic Mapping Tools program [25].

The Hammerschmiede outcrop is an active clay-pit in the Upper Series lithostratigraphic unit of the Upper Freshwater Molasse in the North Alpine Foreland Basin [6, 24] (Fig 1A and 1B). It exposes a more than 25 meters thick fluvial sequence, composed of clayey to silty overbank sediments, incised by sandy channel-fills, in addition to two lignite seams, representing a swamp facies [6]. Fossils are mainly known from fluvial channels. The fossil-bearing level HAM 5 represents a riffle pool sequence of a small meandering rivulet (Fig 1C) [5]. The fossiliferous, one-metre thick channel-fill is composed of three fining-upward beds with reworked pedogenic carbonates at their bases [11]. Skeletal elements of vertebrates are commonly disarticulated and show no abrasion, except for some specimens from large bodied taxa such as rhinos and proboscideans. Associated elements of medium-sized mammals like *Danuvius guggenmosi*, are found frequently within and especially next to the channel (Fig 1C), suggesting rapid deposition and only minor downstream transport of carcasses.

The material described here was recovered from the HAM 5 channel structure in close proximity to the hypodigm of *Danuvius guggenmosi* (Fig 1C). Specimens GPIT/MA/10007 and 13005 were found in 2011 next to each other during the first test excavations in Hammerschmiede. Therefore, we have no tachymeter measurements for those specimens. GPIT/MA/13004 has been excavated in 2017, about 25 m downstream from the first two specimens (Fig 1C).

## Materials and methods

### Comparative material

GPIT/MA/13005, 13004, and 10007 were compared to fossil catarrhines (propliopithecoids, pliopithecoids, cercopithecoids, hominoids) and to extant catarrhines. Morphometric data on extant catarrhine patellae have been taken from zoological collections of the Royal Museum for Central Africa (Tervuren) and the Bavarian State Collection (Munich). Dimensions were measured with dial calipers and recorded to the nearest 0.1 mm.

### ROPA (S1 Fig)

Relative occlusal polygon area (ROPA) is the ratio of the area of the polygon defined by the tips of the four principal upper molar cusps over the total crown base area [26]. Measurements were taken from high-resolution images of original fossil and extant specimens with the maximal occlusal surface view oriented normal to the camera focal plane.

### Protocone and paracone angles (S1 Fig)

The protocone angle, reflecting the positions of the paracone and hypocone relative to the protocone, is measured as the occlusal polygon angle with the protocone at its apex. The paracone angle, reflecting the positions of the protocone and metacone relative to the paracone, is measured as the occlusal polygon angle with the paracone at its apex.

### Micro-CT scanning

The teeth were scanned with an X-ray tube containing a multi-metal reflection target with a maximum acceleration voltage of 225 kV in the Nikon X TH 320 μCT scanner of the 3D imaging lab of the University of Tübingen. All specimens (except GPIT/MA/10000-03, for scan data see [5]), were scanned using a 0.1 mm copper filter, but with different settings: GPIT/MA/10001-01 was imaged using 4476 projections, at 210 kV and 45 μA with a voxel size of 0.011504 mm; GPIT/MA/10002-07 with 4476 projections, 180 kV and 50 μA, with a voxel size of 0.011942 mm; and GPIT/MA/13005 with 3500 projections, 200 kV and 27 μA, with a voxel size of 0.006741.

### Calculations of 2D enamel thickness

Following [27], virtual buccolingual sections of the teeth were performed using Dragonfly software, Version 2022.1.0.1259 for Windows (http://www.theobjects.com/dragonfly). Mesial and distal virtual sections in upper molars were defined by the tips of the protocone-paracone and metacone–hypocone. The following variables were measured two-dimensionally in both planes: dentine area (b), enamel cap area (c), length of the enamel–dentine junction (e) and the bi-cervical diameter. According to [28] the average enamel thickness is calculated as c/e and the relative enamel thickness (RET) is calculated as $\frac{\left(\frac{c}{e}\right)}{\sqrt{b}}*100$.

The investigated molars show different stages of wear [29], ranging between unworn (wear stage 1) and full cusp removal with some to large dentine exposure (wear stage 3–4). For worn molars (M^1^, M^2^ from *Danuvius guggenmosi* holotype maxilla and the paratype M^1^, see [5]) we performed measurements on digitally reconstructed enamel surfaces.

### Nomenclatural acts

The electronic edition of this article conforms to the requirements of the amended International Code of Zoological Nomenclature, and hence the new names contained herein are available under that Code from the electronic edition of this article. This published work and the nomenclatural acts it contains have been registered in ZooBank, the online registration system for the ICZN. The ZooBank LSIDs (Life Science Identifiers) can be resolved and the associated information viewed through any standard web browser by appending the LSID to the prefix ""http://zoobank.org/"". The LSID for this publication is: urn:lsid:zoobank.org:pub:78A8F228-A9BB-4A5D-9D1E-2BD733BF1DE8. The electronic edition of this work was published in a journal with an ISSN, and has been archived and is available from the following digital repositories: PubMed Central, LOCKSS.

## Results

### Systematic paleontology

Order Primates Linnaeus, 1758

Infraorder Catarrhini Geoffroy, 1812

Family Hominidae Gray 1825

***Buronius manfredschmidi* nov. gen. et sp**.

***urn:lsid:zoobank.org:act:C21EDA4A-F916-40CE-98FE-528AC82317F9***.

#### Derivation nominis

Genus name after *Buron*, the medieval name for the city of Kaufbeuren, which is located 5 km to the south of the Hammerschmiede clay-pit. The specific epithet is in honour of Dr. med. dent. Manfred Schmid (Marktoberdorf), a private collector who joined Sigulf Guggenmoos in collecting fossils from Hammerschmiede since the late 1970’s.

#### Holotype

**Fig 2:**
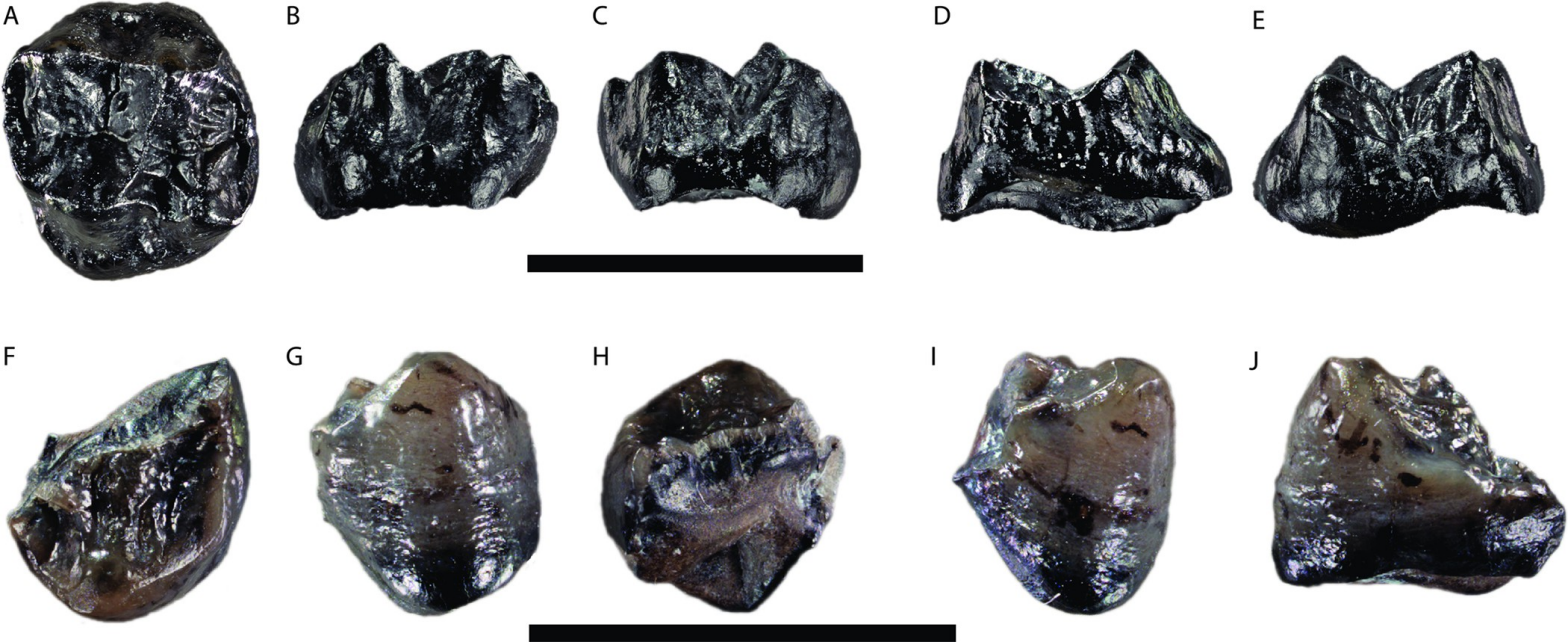
*Buronius manfredschmidi* nov. gen. et sp. photographs. Upper panel: holotype left upper M^2^ (GPIT/MA/13005), A–occlusal, B–buccal, C–lingual, D–mesial, E–distal. Lower panel: paratype left lower P_4_ (GPIT/MA/13004), F–occlusal, G–buccal, H–lingual, I–mesial, J–distal. Scale bars equal 10 mm.

**Fig 3:**
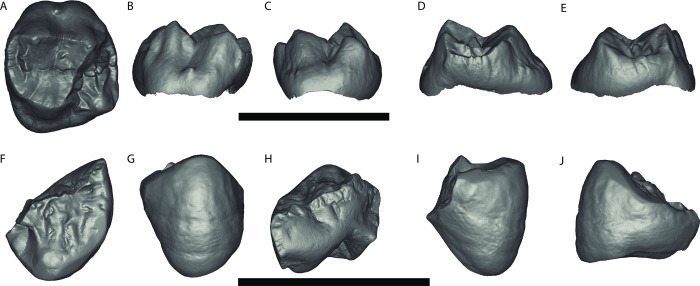
*Buronius manfredschmidi* nov. gen. et sp. surface renderings. Upper panel: 3D rendering of the holotype left upper M^2^ (GPIT/MA/13005), A–occlusal, B–buccal, C–lingual, D–mesial, E–distal. Lower panel: 3D rendering of the paratype left lower P_4_ (GPIT/MA/13004), F–occlusal, G–buccal, H–lingual, I–mesial, J–distal. Scale bars equal 10 mm.

GPIT/MA/13005: An unworn left upper M^2^ germ, crown complete with no root formation (Figs 2 and 3).

#### Paratypes

**Fig 4:**
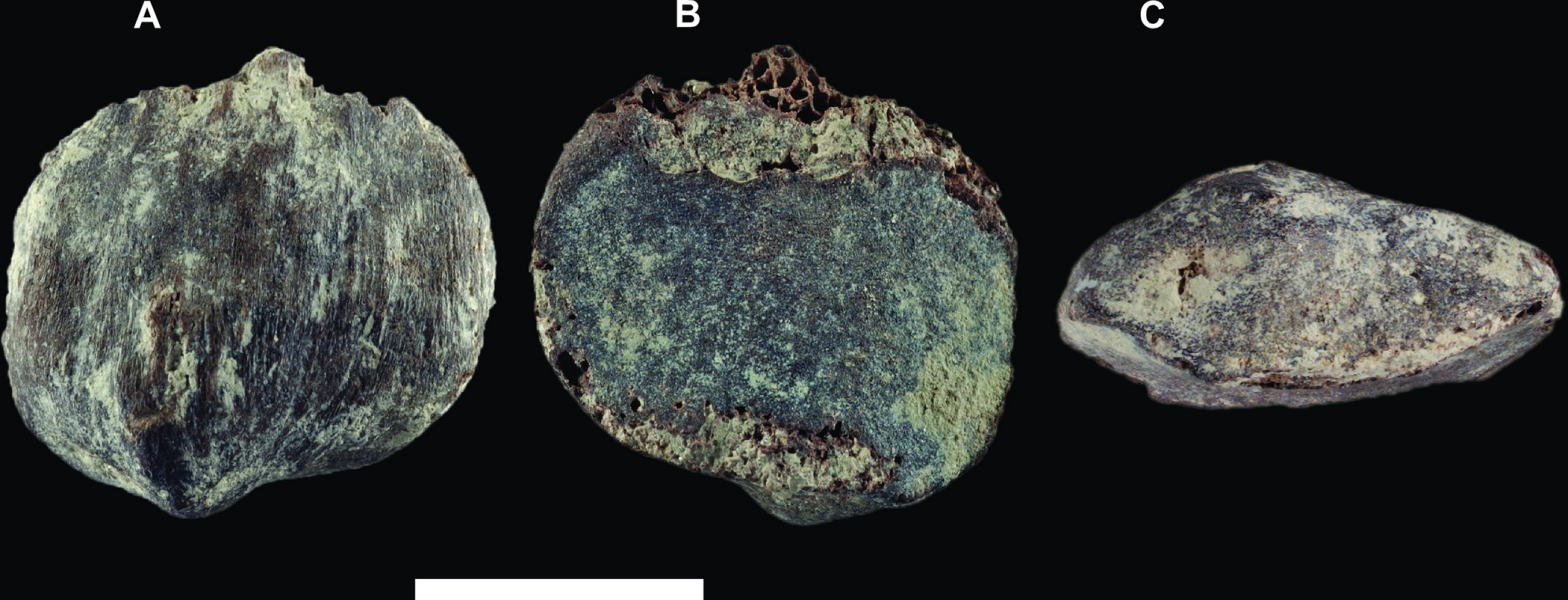
*Buronius manfredschmidi* nov. gen. et sp. paratype left patella (GPIT/MA/10007). Anterior (A), posterior (B) and distal (C) views. Scale bar equals 10 mm.

GPIT/MA/13004: A lightly worn left lower P_4_ fragment preserving the buccal portions including the mesial fovea, an intact protoconid, and the buccal half of the talonid and metaconid, and missing the lingual third of the crown (Figs 2 and 3). GPIT/MA/10007: A left patella slightly damaged proximally, with a porous articular surface suggestive of a juvenile (Fig 4 and S2 Fig).

Measurements of the holotype and paratypes are provided in Table 1.

**Table 1:** *Buronius manfredschmidi* nov. gen. et sp., tooth and patella metrics (mm).

Collection number	tooth position	MD	BLm	BLd	H
GPIT MA/13004	Lp4	5,9	?	?	5,7
GPIT MA/13005	LM2	7,7	8,6	8,2	5,0
		PDAS	PD	AP	ML
GPIT MA/10007	left patella	15,0	16,6	7,2	16,6

MD—mesio-distal length, BL—bucco-lingual breadth, m—mesial, d—distal, H–height, PDAS—proximo-distal articular surface length, PD—proximo-distal length, AP—anterior-posterior thickness, ML—medio-lateral breadth.

#### Locality and horizon

Hammerschmiede Clay pit near Pforzen (Allgäu region, Bavaria, Germany; 47.927° N, 10.592° E); level Hammerschmiede (HAM) 5 at stratigraphic metre 12 in the local section, which has been dated magnetostratigraphically to 11.62 million years ago [6].

#### Diagnosis

Small hominid in the low end of the body size range of *Symphalangus*, suggesting an average body mass of about 10 kg. **M**^**2**^ with prominent cusps positioned near the periphery of the crown, with protocone-paracone and hypocone-metacone nearly aligned transversely, a shallow lingual cingulum confined to the protocone, broad mesial fovea, mesiodistally long trigon, lingually placed metacone and hypocone, well developed and continuous crista obliqua, slit-like distal fovea, hypocrista well elevated in relation to the distal marginal ridge, thinly enamelled with prominent, widely spaced dentin horns, and sloped, concave (with respect to the dentine) lingual enamodentin junction. **P**_**4**_ with protoconid and metaconid of equal prominence, moderate protoconid lingual flare, well developed protocristid elevated relative to the thick mesial marginal ridge, broad, deep mesial fovea, elongated trigonid with a strongly inclined distolingual margin, modestly developed hypoconid, sharp distal marginal ridge with multiple conulids. **Patella** oblique ovoid in shape, relatively thick and broad compared to length, well developed keel separating relatively concave condylar surfaces resulting in a saddle shaped joint surface, prominent and medially shifted distal apex.

#### Differential diagnosis

**Fig 5:**
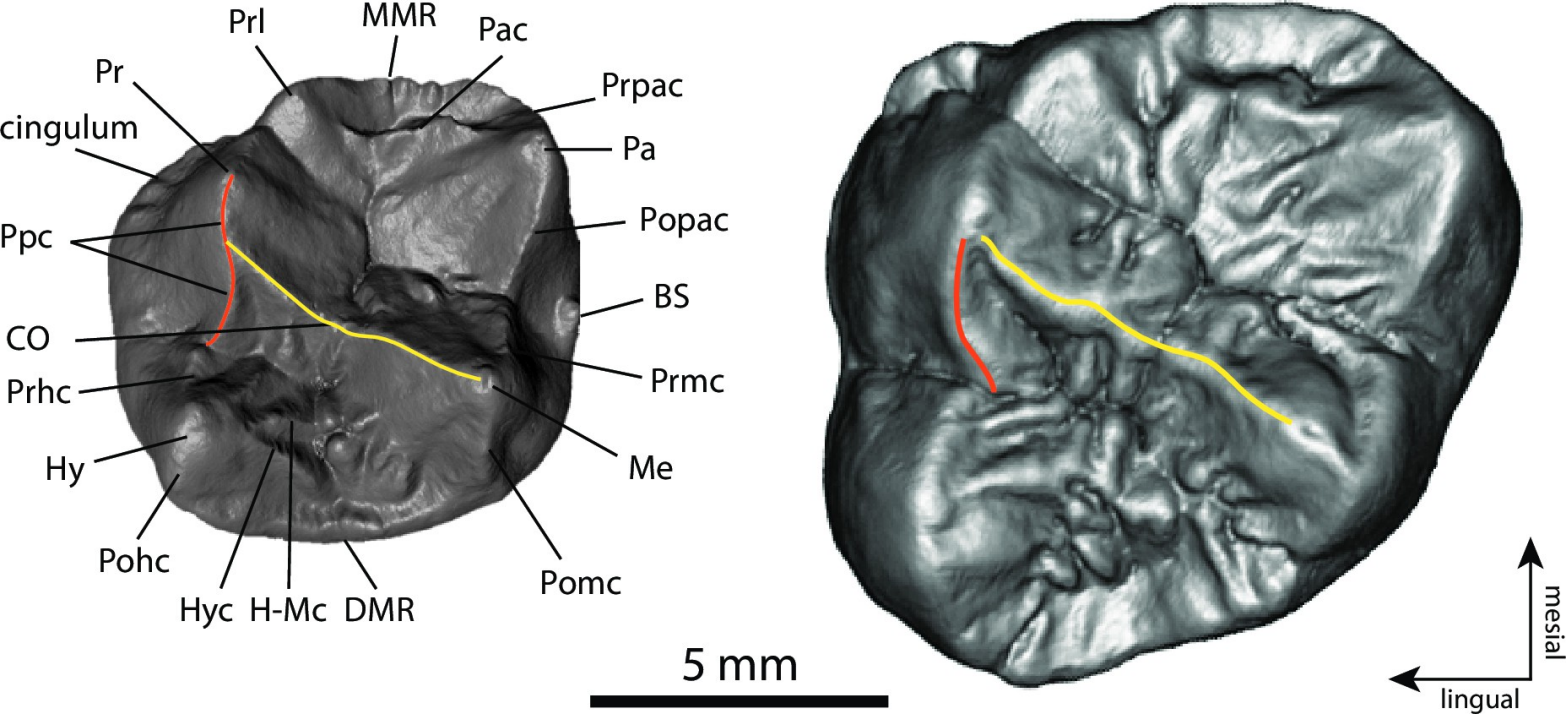
Comparison of *Buronius* (left, GPIT/MA/13005) and *Danuvius* (right, GPIT/MA/10002-07) upper M^2^ occlusal morphology with the nomenclature labelled on the *Buronius* tooth. Pr—protocone; Prl—protoconule; MMR—Mesial marginal ridge; Pac—paracrista; Prpac—preparacone crista; Pa—paracone; Popac—postparacone crista; BS—buccal style; Prmc–premetacone crista; Me—metacone; Pomc—postmetacone crista; DMR–Distal marginal ridge; H-Mc–hypocone-metacone crista; Hyc—hypocrista; Pohc—posthypocone crista; Hy—hypocone; Prhc—prehypocone crista; CO—crista obliqua; Poprc—postprotocone crista. Note the unusual configuration of the crista obliqua (yellow line) and postprotocone crista (orange line). In *Buronius* the crista obliqua meets the concave postprotocone crista distal to the protocone, in contrast to *Danuvius*, in which the two cristae meet at the protocone tip (For a description of GPIT/MA/10002-07 see S2 File).

*Primitive catarrhines*, *early Miocene stem hominoids*, *and middle Miocene stem hominids*. GPIT/MA/13005 (Figs 2, 3, 5, 8 and S3, S4, S7, S8 Figs and Table 1) differs from *Griphopithecus* in being much smaller and in having thin enamel. It differs from early Miocene hominoids (e.g. *Ekembo*) in being smaller than all but the smallest specimens, reduced lingual cingulum, longer relative to breadth, having expanded mesial fovea and elevated protocrista and hypocrista relative to the mesial and distal marginal crista. Differs from *Pliobates* in being larger, elongated relative to breadth, reduced lingual cingulum, larger mesial and distal fovea, and reduced buccal style. Differs from *Anapithecus* and other pliopithecoids in being longer relative to breadth, reduced lingual cingulum and buccal style, broader more peripheralized cusps, less compressed and elevated cristae, larger mesial fovea, larger, elongated trigon, lower mesial and distal marginal ridges. Differs specifically from *Anapithecus* in much less strongly developed cingulum, which is better described as a rounded rim rather than a true cingulum (lacking a sharp margin, the shelf between the margin and crown wall, and the cristae between the crown wall and the cingulum edge), greater cusp peripheralization, more lingually inclined postparacrista, postparacrista-premetacrista not aligned and positioned closer to the buccal margin of the crown, shorter talon, slit-like distal fovea lacking the distal expansion seen in *Anapithecus*, hypocone more cone-shaped (in comparison with the pointed hypocone in *Anapithecus*), positioned at the distolingual corner of the crown, mesially oriented prehypocone crista connected to the postprotocone crista (which is absent in *Anapithecus*) rather than in crista obliqua. The preparacone cristae also differ in being sharper and more directly aligned with the other buccal cristae in *Anapithecus*, while strongly curved mesiolingually and less sharp in *Buronius*.

GPIT/MA/13004 (Figs 2 and 3 and Table 1) differs from *Ekembo*, *Proconsul*, *Afropithecus*, *Griphopithecus*, *Equatorius*, *Kenyapithecus*, and *Nacholapithecus* in the height of the talonid relative to the trigonid.

GPIT/MA/10007 (Fig 4 and S2 Fig and Table 1) differs from stem hominoids (*Ekembo*, *Equatorius*, *Nacholapithecus*) and *Epipliopithecus* in being proximo-distal shorter and antero-posteriorly thicker.

*Middle/late Miocene hominids/hominines*. GPIT/MA/13005 (Figs 2, 3, 5, 8 and S3, S4, S7, S8 Figs and Table 1) differs from *Danuvius* in being much smaller and in having a more strongly developed protocone lingual cingulum, relatively larger metacone, more sloped lingual and buccal crown surface, larger mesial fovea, more strongly developed paracrista connected to the protoconule, elevated hypocrista relative to the distal marginal ridge, more elongated trigon, less truncated crown shape distobuccally, a more strongly developed buccal style conule, more peripheralized cusps and thinner enamel (possibly except *Dryopithecus*, see discussion). Differs from *Pierolapithecus*, *Anoiapithecus* and *Dryopithecus* in being smaller and in the development of the mesiolingual cingulum, metacone size, crista development, and enamel thickness. Differs from *Rudapithecus* and *Hispanopithecus* in being smaller and in having more a strongly pronounced mesiolingual cingulum, a larger mesial fovea, a more prominent hypocrista and more sloped lingual and buccal surfaces. Quantitatively, *Buronius* is much smaller than any dryopithecin M1 and M2 (S5 Fig), has more peripheralized cusps (higher ROPA) than all dryopithecins (Fig 10), a smaller protocone angle than *Danuvius* and a larger protocone angle than most *Hispanopithecus* (Fig 9C), a smaller paracone angle than *Anoiapithecus*, *Dryopithecus* and *Rudapithecus*, and a larger paracone angle than *Danuvius* (Fig 9D). GPIT/MA/13005 is much longer relative to width than the M1 in *Anoiapithecus*, *Dryopithecus* and at the high ends of the ranges of variation in other dryopithecins (S5C Fig). It is longer relative to width than the M2 in *Danuvius* and *Dryopithecus* and at the low ends of the ranges in other dryopithecins (S5D Fig). A PCA also distinguishes *Buronius* from all dryopithecins on component 1, most strongly influenced by protocone angle (Fig 9C). S3 and S4 Figs includes visual comparisons of the occlusal morphology of *Buronius* and dryopithecins and compares GPIT/MA/13005 with associated M1-M2 in other dryopithecins.

GPIT/MA/13004 (Figs 2 and 3; Table 1) differs from dryopithecins (unknown for *Danuvius*) in the elongated talonid and thick mesial marginal ridge.

GPIT/MA/10007 (Figs 4, 11 and S2 Fig and Table 1) differs from *Danuvius* in being smaller, thicker and narrower, with a more strongly developed saddle-shaped joint surface and a prominent distal apex; Differs from *Rudapithecus* and *Pierolapithecus* in its smaller size and prominent distal apex.

### Description

**Fig 6:**
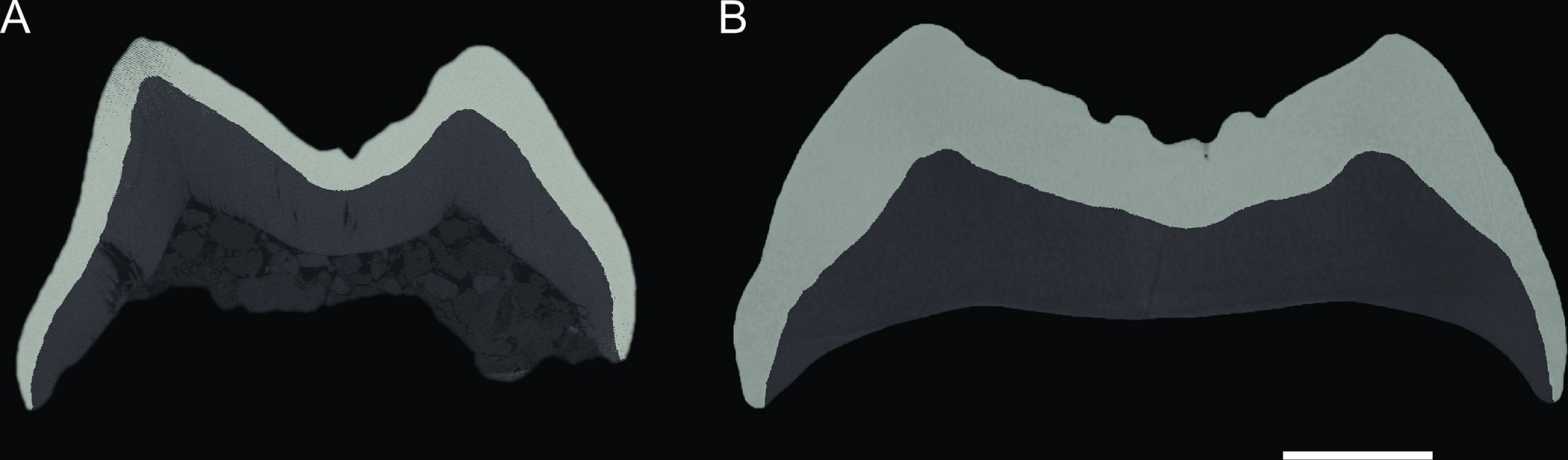
Enamel thickness on unworn upper molars. Distal virtual section through the tips of metacone and hypocone for the left upper M^2^ of *Buronius manfredschmidi* nov. gen. et sp. (A, GPIT/MA/13005) and the left upper M^2^ of *Danuvius guggenmosi* (B, GPIT-MA-10002-07). Note the substantial difference in enamel thickness between *Buronius* and *Danuvius*. Scale bar equals 2 mm.

GPIT/MA/13005 (Figs 2, 3, 5 and S3, S4 Figs) is a perfectly preserved upper M^2^ crown with no wear and no root formation. Crown formation is practically completed and only a very narrow strip on the lingual cervix remains incomplete. The enamel surface is comparatively smooth and few crenulations appear only in the talon basin. The enamodentin junction is well preserved and visible on the underside of the enamel cap. The dentine horns are well separated and compressed at their tips, penetrating deeply into the enamel cap, as confirmed by μct scan (Fig 6). The 2D relative enamel thickness (RET) is quite thin compared with that of *Danuvius* permanent and deciduous dentition (Table 2 and S6 Fig). The protocone is the largest cusp, followed by the paracone and slightly smaller metacone and hypocone, the latter only minimally smaller than the former. The hypocone is lingually positioned relative to the protocone. The metacone has a sloped distobuccal corner. The size and position of the hypocone and the morphology of the metacone are consistent with an M^2^. The trigon is large, especially mesiodistally, with a well-defined paracrista close in height to the mesial marginal crista, separating the trigon from a broad mesial fovea and connected to a well-defined protoconule lingually. The crista obliqua is sharply defined and continuous, with only a shallow notch separating the protocone and metacone portions. The lingual cingulum is shallow and confined to the mesiolingual corner, lacking any development of a shelf or accessory crista, as commonly developed on pliopithecoid upper molars. The hypocrista is strongly developed and elevated in relation to the distal marginal crista. The postmetacrista is equal in length to the premetacrista, while in *Danuvius* the premetacrista is shorter. Buccally there is a mild shelf (style) between the paracone and metacone with a small conule. A peculiarity of GPIT/MA/13005 is the generally concave shape of the cristae, especially the postprotocone crista and the postparacone-premetacone cristae. In most middle and late Miocene European apes these cristae are straight or convex. The concavity of the postprotocone crista is most noticeable (Fig 5). The crista obliqua joins with the postprotocone crista buccal and distal to the protocone, while in *Danuvius* and most dryopithecins it connects directly to the protocone (Fig 5).

**Table 2:** 2D relative enamel thickness of the holotype molar of *Buronius manfredschmidi* nov. gen. et sp. and the molars of the hypodigm of *Danuvius guggenmosi*.

	Tooth	wear stage	Section	c / mm^2^	e / mm	AET / mm	b/ mm^2^	bi-cd / mm	RET
***Buronius manfredschmidi nov*. *gen*. *et sp*.**									
GPIT/MA/13005	M2	1	distal	6,86	14,05	0,49	22,32	8	10,33
	M2	1	mesial	6,2	13,54	0,46	17,74	7,71	**10,87**
***Danuvius guggenmosi***									
GPIT/MA/10000-03	m2	1–2	distal	16,851	19,024	0,89	20,944	7,1	**19,36**
	m2	1–2	mesial	14,667	21,493	0,68	18,115	9,67	16,03
GPIT/MA/10001-01	M1	2	distal	11,71	14,17	0,83	19,07	10,02	18,92
	M1	2	mesial	9,94	12,87	0,77	15,43	9,78	**19,66**
GPIT/MA/10000-01	M1	3–4	distal	15,72	16,9	0,93	33,16	11,22	**16,15**
	M1	3–4	mesial	14,63	16,2	0,90	34,65	11,04	15,34
	M2	3	distal	20,4	17,28	1,18	35,84	11,56	**19,72**
	M2	3	mesial	19,85	18,97	1,05	40,64	13,24	16,41
GPIT/MA/10002-07	M2	1	distal	17,05	15,05	1,13	27,36	10,63	**21,66**
	M2	1	mesial	17,44	15,79	1,10	29,66	11,73	20,28
GPIT/MA/10002-04	dP4	3	distal	5,73	10,17	0,56	7,57	6,67	**20,48**

The values for GPIT/MA/10000-03 are from [5] and wear stage categories from [29]. Abbreviations: c, enamel cap area; e, length of enamel-dentine junction; AET, average enamel thickness; b, dentine area; bi-cd, bi-cervical diameter; RET, relative enamel thickness. Bold font indicates the highest values obtained from a given tooth.

GPIT/MA/13004 (Figs 2 and 3) is a fragmentary left lower P_4_ with light wear along the buccal edge, the buccal and mesiobuccal aspects of the protoconid, and the cusp tip, but without dentin exposure. The roots are not preserved except for a small portion mesially. The metaconid is broken a bit buccal to the apex. The break reveals a thick mesial marginal ridge, also visible in cross section as a prominent enamel fold, with the mesial part thicker than the distal part (Figs 2 and 3). The protocristid is sharp, prominent and well above the mesial marginal ridge. It appears to connect directly to the metaconid. The mesial fovea is deep and probably longer than wide, though it is damaged. The talonid is high in relation to the mesial cusps and the buccal edge from the protoconid to the buccal edge of the talonid (postprotoconid cristid) slopes gradually to the distobuccal margin of the talonid. The cusps are positioned mesially, resulting in an elongated talonid. The long postprotoconid cristid constitutes the buccal and distobuccal edge of the talonid. It curves distolingually to close to the distal margin of the tooth. Unfortunately, there is no P_4_ for *Danuvius* to compare with GPIT/MA/13004.

GPIT/MA/10007 (Fig 4 and S2 Fig) is a small left patella close in size to that of *Symphalangus* and *Ekembo heseloni*. The posteroproximal edge is damaged, more so medially, but this does not significantly affect the measurements. The cortical bone is thin and poorly mineralized, with a porous articular surface and prominent nutrient foramina on the joint surface distally. Cancellous bone is visible on the damaged edge. The fragile preservation suggests that this patella may derive from a subadult individual, while the well-defined edges of the joint surface suggest that adult size had been attained. The patella has an oblique ovoid shape (major axis proximolateral to distomedial), with the joint surface for the lateral condyle superiorly placed relative to the medial surface (Fig 4). However, the maximum proximodistal and mediolateral dimensions are the same (16.6 mm). The lateral border is more convex than the medial border, which is nearly flat around the midline. The articular surface for the femoral patellar groove is well-developed, mediolaterally convex and proximodistally concave (saddle-shaped). The lateral articular facet is wider than the medial one and encompasses about two thirds of the mediolateral breath (Fig 4C). The anterior surface is convex with the same asymmetry as the posterior surface. The highest point of the anterior surface convexity is medially positioned and continuous with the well-developed distal apex, which protrudes distally medial the midline. The apex is a prominent, drop-shaped point (mediolaterally narrow and proximodistally short). The distal apex merges with the distal edges on either side asymmetrically, with the edge being deeper and more strongly notched laterally.

### Taxonomic results

**Fig 7:**
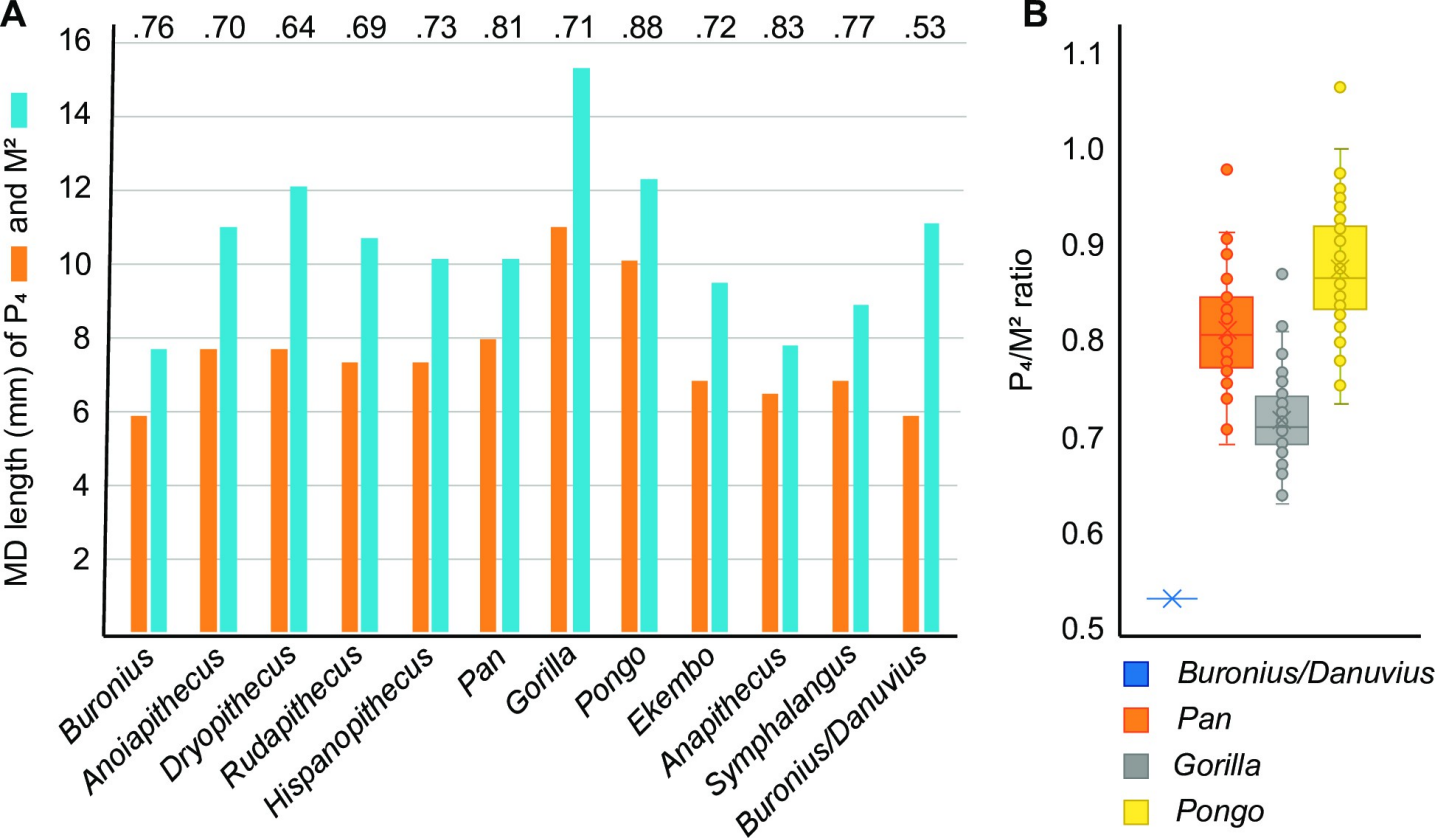
The P_4_/M^2^ ratio. A–Comparison of lower P_4_ (orange bars) and upper M^2^ (blue bars) lengths in selected catarrhines. The relationship between these two teeth in *Buronius* is consistent with other catarrhines. Numbers at top are percentages of premolar to molar length. Note the much lower ratio of the *Buronius* P_4_ and the smallest *Danuvius* M^2^. B–P_4_/M^2^ within individual ratios in great apes compared with the same ratio between *Buronius* P_4_ and the smallest *Danuvius* M^2^. The box plot shows the centre line (median), box limits (upper and lower quartiles), crosses (arithmetic mean), whiskers (range) and individual values (circles).

Based on tooth size alone (both *Buronius* teeth are consistent in size with a single species, Fig 7A), *Buronius* is unlikely to be a small *Danuvius* as the difference in tooth size with the latter exceeds that documented for any catarrhine taxon analyzed here (Fig 7 and S5 Fig). Morphologically, *Buronius* is also quite distinct from *Danuvius* (Fig 5) and differs in numerous characters, as in a more sloped lingual and buccal crown surface, in a better development of protocone lingual cingulum and paracrista, the latter connected to the protoconule, by both larger mesial fovea and metacone, by a more elongated trigon, and an elevated hypocrista relative to the distal marginal ridge, by a less truncated crown shape distobuccally, a more strongly developed buccal style conule, by more peripheralized cusps and finally by thinner enamel.

**Fig 8:**
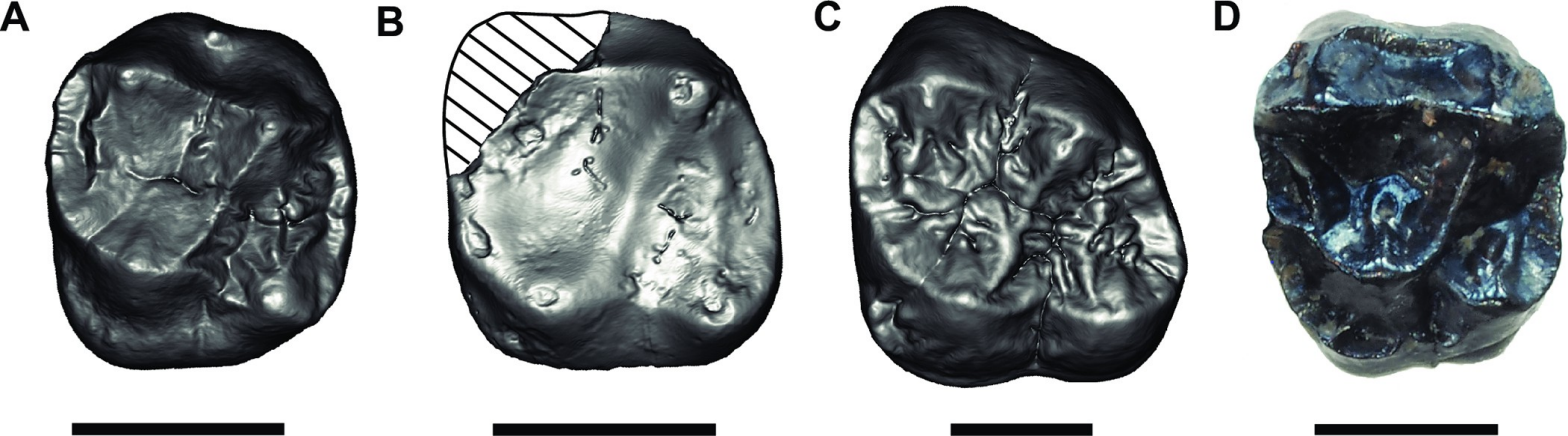
The *Buronius* M^2^ (A) compared with a dP^4^ (B), and M^2^ (C) of *Danuvius* and a M^2^ of *Anapithecus* (D). (A–GPIT/MA/13005; B–GPIT/MA/10002-04, inverted; C–GPIT/MA/10002-07; D–RUD 90). Scale bars equal 5 mm. Mesial is to the left.

**Fig 9:**
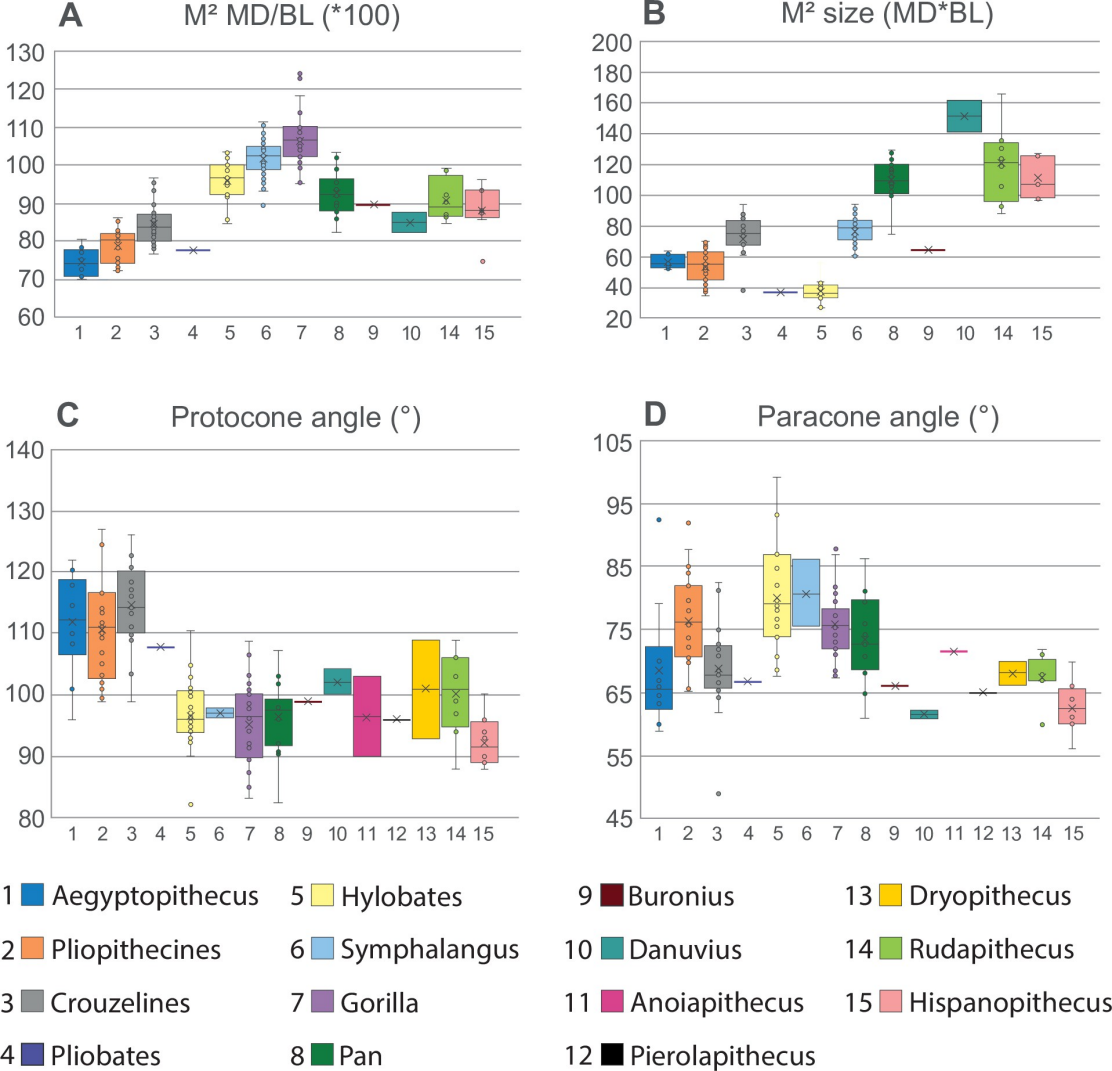
Comparative analysis of the *Buronius* upper M^2^. A-D–Box plots of length/breadth ratios, molar size and protocone and paracone angles. *Buronius* falls among fossil and living apes in length/breadth and among larger primitive catarrhines and siamangs in overall size. The protocone angle (see Methods) clearly distinguishes *Buronius* from primitive catarrhines including *Pliobates*. Curiously, in paracone angle living apes are distinct from both primitive catarrhines and fossil great apes including *Buronius*. All box plots show the centre line (median), box limits (upper and lower quartiles), crosses (arithmetic mean), whiskers (range) and individual values (circles). C—Principal component analysis based on mesiodistal length, buccolingual breadth, ROPA, protocone and paracone angles in *Buronius* and dryopithecins. *Hispanopithecus* (green dots) and *Rudapithecus* (blue crosses) are mostly distinguished, with a small area of overlap. *Buronius* is isolated from these larger samples. It is also quite distinct from *Anoiapithecus* (red squares), *Pierolapithecus*, (brown inverted triangle), *Dryopithecus* (black circle), and the dryopithecin indet molars from Melchingen (magenta triangle). With larger samples from *Buronius* there may be less of a distinction from *Danuvius*. PC 1 is most strongly influenced by protocone angle and PC 2 by ROPA and paracone angle.

GPIT/MA/13005 is also close in size to GPIT/MA/10002-04, a right deciduous dP^4^ of *Danuvius guggenmosi* (Fig 8B and S7, S8 Figs). However, GPIT/MA/10002-04 is clearly a deciduous molar (low, flared crown, simple occlusal morphology) with thick enamel similar to permanent molars (Table 2 and S6 Fig), and as such is easily distinguished from GPIT/MA/13005. As in permanent molars of *Danuvius*, the preprotocone crista is short and the crista obliqua connects directly to the protocone, but a postprotocone crista is absent in the deciduous molar of this species (Fig 8). We describe and document the differences between upper deciduous last molars and GPIT/MA/13005 in the Supporting Information file (S7–S14 Figs and S1 Table). GPIT/MA/13005 is narrower than pliopithecoids M2s, and closest to the means of *Pan*, *Rudapithecus* and *Hispanopithecus* (Fig 9A). The protocone angle in GPIT/MA/13005 is lower than in pliopithecoids, propliopithecoids and *Pliobates*, and within the 25–75% quartiles of most extant hominoids and dryopithecins (Fig 9C). This reflects in large part the position of the hypocone relative to the protocone, with a more lingually displaced hypocone associated with larger angles. Paracone angles, which reflect the position of the metacone relative to the paracone, distinguish less between primitive catarrhines and hominoids, but do separate *Buronius* and *Danuvius* (Fig 9D).

**Fig 10:**
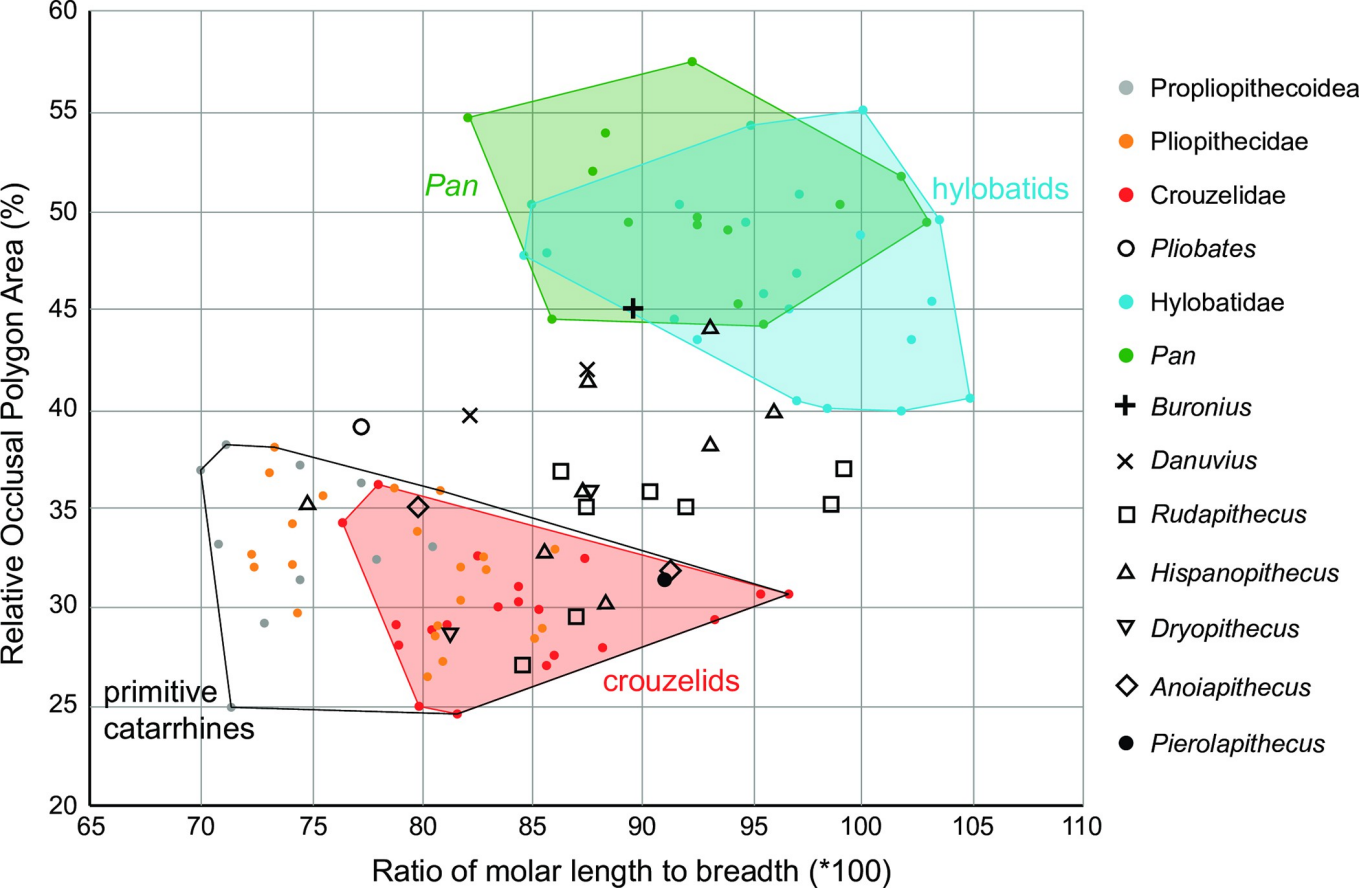
Relative Occlusal Polygone Area (ROPA). The ratio of M2s mesiodistal/buccolingual dimensions (X axis) relative to ROPA (see Methods section and S1 Fig). Primitive catarrhines (including *Pliobates*) separate well from extant hominoids, with many fossil great apes falling between the two groups. *Buronius* falls in the overlap of the hylobatid and *Pan* polygons.

GPIT/MA/13005 differs in occlusal morphology from all early and middle Miocene catarrhines and most closely resembles that of late Miocene apes. The M^2^ is unlike pliopithecoids and *Pliobates*, in having a reduced, smooth cingulum and buccal style and being narrower relative to length (Figs 8 and 9A). Crista are less strongly developed, cusps less compressed and basins broader and shallower. The cingulum and style distinguish it from *Danuvius* and all European hominines (several recent comprehensive phylogenetic analyses differ in their placement of some European late Miocene apes as stem hominines or stem hominids; for a more complete discussion of middle and late Miocene ape phylogeny see [30–33] Cusp peripheralization, as measured by the relationship of total crown basal area (TCBA) compared with occlusal polygon area (Fig 10) groups *Buronius* with extant hominoids and far from primitive catarrhines. The P_4_ fragment lacks the typical pliopithecoid cristodonty and deep basins.

To summarize, while GPIT/MA/13005 is in the size range of larger, stratigraphically younger pliopithecoids, it is easily distinguished from them in morphology. GPIT/MA/13005 clusters with hominoids but without a specific similarity to *Danuvius* or other late Miocene apes. The distinction from *Danuvius* is especially pronounced in overall size (Fig 9B and S5 Fig) and in the comparison of enamel thickness, which is among the thickest for late Miocene apes in *Danuvius* and the thinnest for *Buronius* (Table 3). GPIT/MA/13005 is unique in its combination of size and morphology, which justifies a new genus. We acknowledge that the presently known hypodigm is small. Larger samples are needed for a more complete characterization of *Buronius*.

**Table 3:** 2D relative enamel thickness (RET) in *Buronius* and fossil and extant hominoids.

Taxon	sample size	Tooth	RET
*Buronius*	1	M^2^	10.33–10.87^1^
*Danuvius*	2	M^2^	16.41–21.46^1^
	2	M^1^	15.34–19.66^1^
*Dryopithecus*	1	M^2^	12.5
*Pierolapithecus*	1	M^2^	14.4
	1	M^1^	14.0
*Anoiapithecus*	2	M^2^	13–16.2
	1	M^1^	13.9
*Hispanopithecus*	1	M^2^	14.3
	2	M^1^	12.7–13.4
*Rudapithecus*	1	M^2^	13.5
	1	M^1^	11.29
*Sivapithecus*	2	M^1^	16.3–18.2
*Equatorius*	1	M^2^	20.4
	1	M^1^	17.7
*Griphopithecus*	2	M^2^	17.8–20.2
*Ekembo*	2	M_3_	16.7–17.0
*Pongo*	19	M^2^	11.2–19.2
	24	M^1^	9.0–16.3
*Pan*	~23^2^	M^2^	10.7–12.5
		M^1^	8.5–13.1
*Gorilla*	~13^2^	M^2^	8.9–13.0
		M^1^	7.0–10.2

Measurements were taken on M^2^ and M^1^ except for *Ekembo* (M_3_). *Buronius* is for M^2^ below the ranges of all but *Pan* and *Gorilla*. Data are from this study and Smith et al. [34]. Note 1. Ranges reflect two measurements (mesial and distal) on the same specimens (see Table 2 for individual values). Note 2. Sample size is not specified in the original publication for individual tooth positions. The numbers given here are the presumed total of first and second molars.

**Fig 11:**
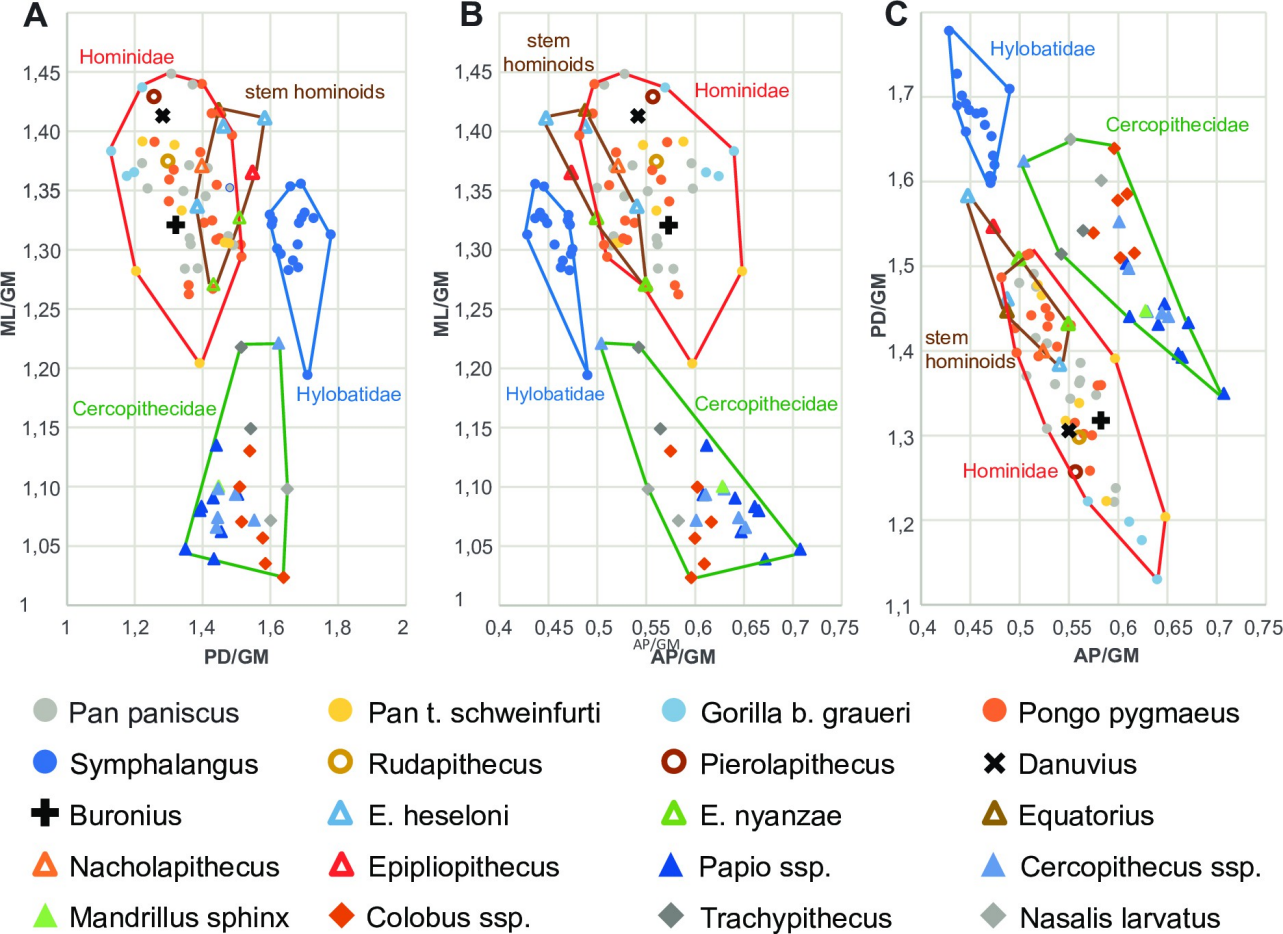
Size-adjusted morphometrics of the patella of extant and fossil catarrhines. GM–geometric mean, ML–mediolateral breath, PD–proximodistal length, AP–anteroposterior thickness. Measurements of fossil stem-hominoids and *Epipliopithecus* are from [37, 38]. Note that the patella morphospace separates the families Cercopithecidae, Hylobatidae and Hominidae. Stem-hominoids (*Ekembo*, *Equatorius*, *Nacholapithecus*) overlap with hominids in the morphospace direction of hylobatids, e.g. they tend to be more elongate and thinner than most hominids.

**Fig 12:**
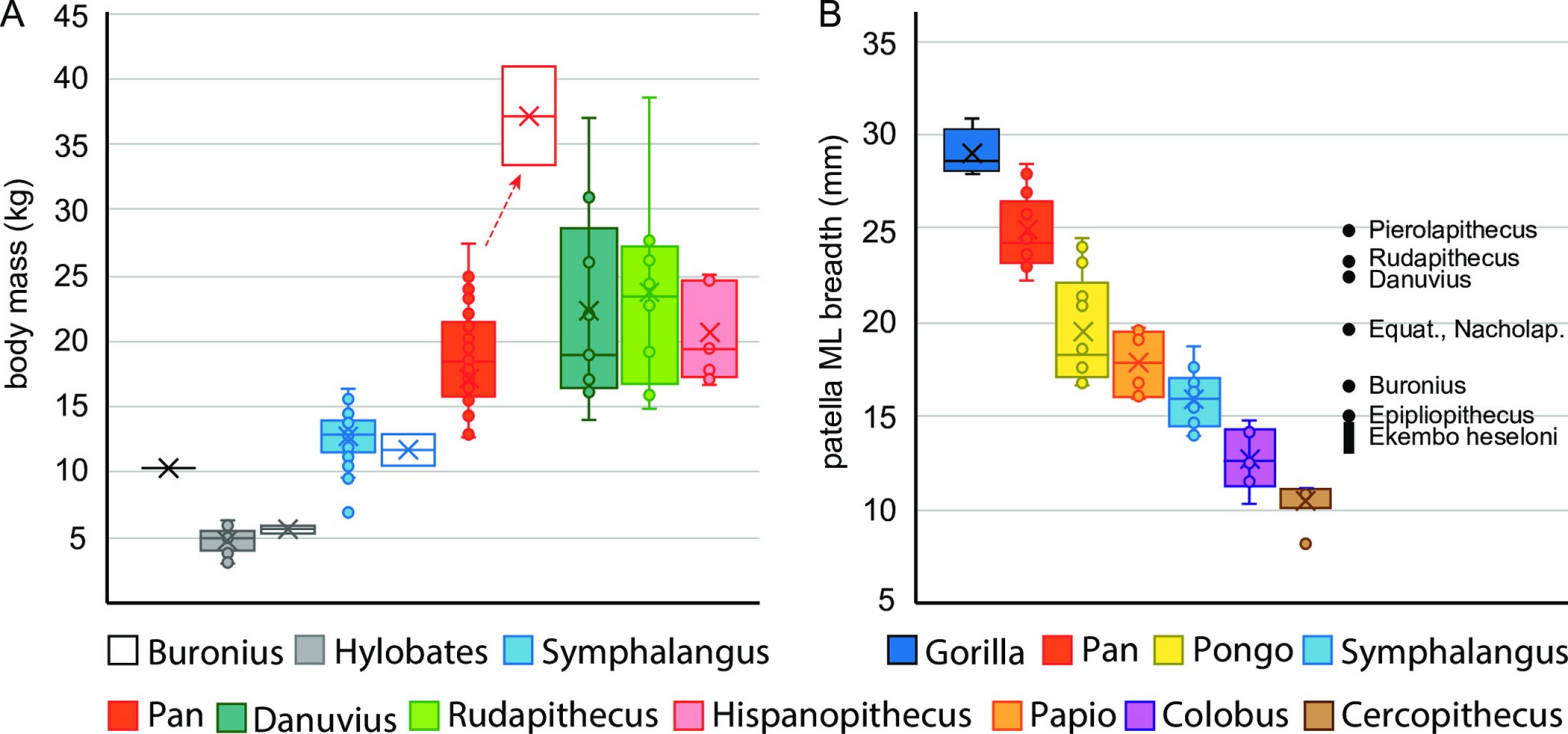
Body mass and patella size. A–Body mass data for extant hominoids, *Buronius*, *Danuvius* and *Rudapithecus* from regressions of M^2^ size [42] and observed body masses (open boxes [43, 44]. Note the close correspondence in hylobatids of values from molar size and observed ranges, in contrast to *Pan*, in which dentally derived estimates of body mass significantly underestimate observed body mass (arrow). The same pattern characterizes *Danuvius* body mass estimates from dental vs postcranial dimensions [5] and Grabowski pers. comm. B–Patellar mediolateral breadth of living catarrhines and selected fossil hominoids (fossil data from [36–38]). *Ekembo heseloni* specimens are from KPS at Rusinga. Larger patella from Rusinga, tentatively attributed to *Ekembo nyanzae* (not shown here) fall among *Danuvius* and *Rudapithecus*. Whatever the actual body mass of *Danuvius* was, it was much larger than *Buronius*, as are all known fossil and extant hominids.

The GPIT/MA/10007 patella is morphologically distinct from the patella attributed to *Danuvius* (GPIT/MA/10000-12) and within the size range consistent with the size of the teeth attributed to *Buronius* (Figs 11 and 12 and S2 Fig). The simplest explanation is that the distinctive small patella belongs to the same taxon as the distinctive small teeth. Extant catarrhine families are easily distinguished in patellar shape (Fig 11). By its roundish overall morphology and anteroposterior thickness GPIT/MA/10007 compares well with fossil and living hominids (Fig 11A and 11B). Stem-hominoids (e.g. *Ekembo*, *Equatorius*, *Nacholapithecus*) and *Epipliopithecus* (the only pliopithecid patella known [35]) are proximodistally slightly longer and hylobatids and cercopithecids (especially colobines) significantly longer (Fig 11A and 11C). The elongation of the patellae of both latter groups results from an extremely pronounced distal apex, which represents, however, an anteriorly flat, very broad (base of apex is as wide as the patella) and tongue-like projection (see e.g. [36] and S2 Fig), unlike the apex found in GPIT/MA/10007. The patella of *Epipliopithecus* is furthermore anteroposteriorly thin, similar to hylobatids. This elongate and thin pliopithecid patella falls between the convex hulls for hominids and hylobatids and within the area defined by the stem-hominoids *Ekembo*, *Equatorius* and *Nacholapithecus* (Fig 11; [37]).

GPIT/MA/10007 differs from the *Danuvius* male patella (GPIT/MA/10000-12) by several features. The *Danuvius* patella is mediolaterally 35% larger, wider than long, the posterior articulation facet is comparatively flat, the anterior surface less convex and the distal apex is practically absent. Furthermore, the size-adjusted anteroposterior thickness is slightly higher in GPIT/MA/10007. The saddle-shaped condylar surface of GPIT/MA/10007 more closely resembles *Rudapithecus*. The distal apex, at which the patellar ligament inserts, is not well-developed in any extant and fossil hominoid, except in one individual of *Ekembo nyanzae* (KNM-RU 18384). However, this patella (S2G Fig) differs from *Buronius* by a stronger symmetry, marked elongation and much larger size. The asymmetric, oblique ovoid shape with a medially shifted apex may represent unique features of the new genus. A pointed patellar apex is otherwise known from the genus *Homo*, which however is longer (25% of the proximodistal patellar length) with a wider proximal base [39].

Based on patellar and tooth morphology *Buronius* differs from stem-hominoids and hylobatids, having its closest affinities with hominids. We conclude that *Buronius* is most probably a crown hominid (including all living great apes, humans and their fossil relatives).

### Enamel thickness

The unworn upper M^2^ of *Buronius manfredschmidi* exhibits a 2D relative enamel thickness (RET) of 10.87 (Table 2). This value contrasts with the thickly enamelled permanent and deciduous molars of *Danuvius guggenmosi*, which range for six molars between 16.15 and 21.66 (m2 19.36; dP4 20.48; M1 mean 17.9, n = 2; M2 mean 20.69, n = 2; Tables 2 and 3). Table 3 shows the range of RET values in a number of Miocene apes and crown hominids.

## Discussion

The occlusal morphology of GPIT/MA/13005 (M^2^) is perfectly preserved, which makes comprehensive comparisons possible despite the limited sample. Its uniqueness, both in size and morphology, warrants the recognition of a new genus. The size and morphology of GPIT/MA/13004 (P_4_) and GPIT/MA/110007 (patella) are consistent with this conclusion. While the small sample size is less than ideal, what is known of *Buronius* cannot be accommodated within any known hominid taxon. While clearly different from pliopithecoids, the small hominid from HAM 5 is also distinguished from most Miocene apes. Regarding the holotype (GPIT/MA/13005), there is no close match with early and middle Miocene apes in quantitative morphology or in the morphology of the cusps, cristae and basins. The closest morphological match is with late Miocene European apes and extant hominoids. Like late Miocene hominines (or hominids) the cusps in GPIT-MA 13005 are widely spaced, the trigon is spacious and the crista obliqua is sharply defined. Pliopithecoid upper molars are distinguished from hominids in crown shape, cingulum development, and cusp peripheralization. The lower P_4_ is also distinguished from pliopithecoids in cusp and basin morphology. Interestingly, *Buronius* is more modern in cusp peripheralization than most middle and late Miocene hominids, which fall between extant hominids and primitive catarrhines (Fig 10). One specimen of *Hispanopithecus* also falls within the extant polygons. If the distributions of *Rudapithecus* and *Hispanopithecus* are an indication of typical within-genus variation, a larger sample of *Buronius* may help to clarify the distinction from other late Miocene apes.

### Relative enamel thickness

*Buronius* has the lowest RET of any Miocene ape for which data are known [5, 34]. The relative enamel thickness (RET) of the five dryopithecin genera *Anoiapithecus*, *Pierolapithecus*, *Dryopithecus*, *Rudapithecus*, *Hispanopithecus* is higher than in *Buronius* (Tables 3 and 4). RET in *Buronius* is much smaller than in *Danuvius*, with *Danuvius* falling among the most thickly enamelled taxa in our sample. However, sample-sizes for RET of fossil hominids is small. Larger samples of extant great apes, particularly *Pongo*, can show substantial variation in enamel thickness (Table 3). The lowest value measured so far from a European ape is an M^2^ of *Dryopithecus fontani* [40], which is about 20% thicker than in *Buronius* (Table 3), but both values may be included in the variance of the species if larger samples are available. Therefore, it is possible that *Buronius* cannot be differentiated from *Dryopithecus* based on RET alone.

**Table 4:** Relative enamel thickness in extant hominoid upper second M^2^ and their thickness categories and dietary affinities.

Taxon	Diet	M^2^ RET
*Gorilla*	Folivore	10.7 (8.9–13.0)
*Symphalangus*	Folivore	10.8 (9.02–12.65)^1^
*Pan*	Fruit	11.7 (10.7–12.5)
*Hylobates*	Fruit	11
*Pongo*	Hard/tough	14.0 (11.2–19.2)
*Homo*	Omnivore	22.35 (13.76–32.26)
*Buronius*	Fruit/leaves?	10.33–10.87
*Danuvius*	Hard/tough?	19.72–21.46

*Buronius* falls within the range of extant folivores while *Danuvius* falls among hard object feeders/omnivores. We recognize that primate folivores also consume fruit and frugivores consume leaves and animal protein. Whether or not *Buronius* was more folivorous and *Danuvius* more of a hard/tough object feeder, it seems clear that there were pronounced dietary differences between the two, well beyond what would be expected within a single genus. RET values are from [34]. Note: *Symphalangus* data from M_1_, which is closest to M^2^ values in extant hominoids.

The 2D RET value of *Buronius* is closest to the means of gorillas and siamangs while *Danuvius* is in the upper end of the range of variation in *Pongo* and within the range of modern humans, *Sivapithecus*, and *Griphopithecus* (Table 3). The pronounced disparity in enamel thickness between *Buronius* and *Danuvius* strongly supports the genus-level distinction. The difference in enamel thickness between *Danuvius* and *Buronius* is not attributable to the difference often observed between permanent and deciduous teeth, as we have demonstrated that the type of *Buronius* is a permanent upper molar (see above and Supporting Information file, S6–S14 Figs).

### Patella function

The accentuated apex of the GPIT/MA/10007 patella is probably related to the development and orientation of the patellar ligament and quadriceps femoris force transmission. Furthermore, the better expressed convexity of the articular facets implies a deeper patellar groove of the femoral trochlear surface in GPIT/MA/10007 than in *Danuvius*. This suggests a greater degree of constraint on the direction of forces resulting from quadriceps femoris contraction in the former than the latter [36], A relatively short, thick patella, as found in *Buronius*, has been related to the mechanical advantage of the quadriceps, which is apes has been related to climbing [36]. The asymmetry of the patella may reflect differences in the development of the vasti muscles, with a larger vastus lateralis compared with the vastus medialis. The combination of asymmetry, patellar shape and the development of the apex may all be related to a specific pattern of quadriceps femoris function in *Buronius* [36, 41].

The greater extent of the vastus lateralis insertion and its greater distance from the patellar ligament attachment on the patella suggests an emphasis on a response to adduction moments, to maintain the knee in a neutral or abducted position. The asymmetric position of the patellar apex, which influences the position of the patellar ligament, may also reflect quadriceps function. The development of the patellar apex may be related to the force being transmitted thought the patellar ligament or it may serve to maintain the orientation of the ligament relative to the quadriceps and the long axis of the tibia. It, along with the more strongly developed saddle-shaped joint surface suggests some degree of directional constraint to the forces crossing the knee joint. Without additional postcranial elements for *Buronius* it is difficult to reconstruct the precise behavioural implications of this morphology except to say that it probably excludes stereotypical pronograde quadrupedalism. Similarity with *Rudapithecus*, which is known from many postcranial elements with a strong arboreal and suspensory signal, suggests the same for *Buronius*, with perhaps a greater degree of constraint in knee extension.

### Body weight

The teeth and patella of *Buronius* are close in size to siamangs, suggesting a body mass of about 10 kg (Fig 12). In contrast, *Danuvius guggemosi* has a calculated body mass ranging from 17 to 31 kg using regressions for several measurements from the femur and tibia [5] or, by using a different methodology of reconstruction, from 14.5 to 46.3 kg (Mark Grabowski personal communication 27. February 2023).

Fig 12A depicts body mass ranges for a variety of hominoids based on both estimates from M^2^ size and observed body masses [42–44]. Hylobatid body mass ranges are very similar between estimates from dental size and observed masses. This suggests that in this range body mass estimates from M^2^ size in hominoids are reliable. However, in *Pan* body mass estimates from M^2^ size are much lower than actual body masses collected from different individuals (arrow). In Gingerich et al. [42], from which the formula to estimate body mass was taken, the body masses for male and female *Pan* used by the authors to produce the formula (43900g and 31500g respectively) are higher than those predicted by M^2^ size by 53 and 40% respectively. Body mass estimates from dental data for *Pan* underestimate actual mass, but it is not clear if this is specific to *Pan* or an effect of size. A size effect would explain the discrepancy in dental vs postcranial derived estimates for similarly sized *Danuvius*. Either way, the estimate for *Buronius* appears to be reliable as far as body mass estimates from teeth go, and clearly shows that it is much too small to be *Danuvius*. Similarly, the mediolateral breadth of the patella, which scales with body mass [45], falls within the range of siamangs and is near to *Ekembo heseloni*. Until more postcranial specimens of *Buronius* are found, we think that our conservative estimate of a body mass around 10 kg is reliable.

### Hominid sympatry

The taphonomic context of fossils assigned to *Buronius manfredschmidi* and *Danuvius guggenmosi* support the direct sympatry (sensu [46], in contrast to broad sympatry over wider geographic areas), of both species in the immediate habitat of the ecosystem of Hammerschmiede level HAM 5 (Fig 1). To avoid competition, directly sympatric primates have to rely on different resources [46]. The differences between *Buronius* and *Danuvius* in functional tooth morphology, enamel thickness, patellar morphology, and body mass strongly support resource partitioning between both species. The thin tooth enamel with fewer crenulations and the relatively more accentuated shearing crests of the small-sized *Buronius* suggests a more fibrous diet that may have included both soft fruits and more fibrous vegetation. However, there is little indication of specialized folivory, as in gorillas or siamangs, although in enamel thickness *Buronius* is more similar to folivores (Table 4). In contrast, the larger *Danuvius guggenmosi* shows blunter molar cusps and crenulated tooth enamel that is, even in its deciduous dentition, near twice as thick as in *Buronius* (Figs 5 and 6), pointing to higher bite forces and a diet including harder/tougher food items (cf. [47, 48]). The differences in body size and patellar morphology suggest differences in canopy use, with *Buronius* possibly spending more time higher in the canopy, possibly in the terminal branches. However, it is likely that most if not all European hominoids were opportunistic feeders, given the latitudinal and climatic position of their habitats. Even if the winter season was warm, it had a short day-length, implying reduced photosynthesis and more limited availability of fresh leaves and other potential fallback foods. They likely experienced selection to exploit a wide range of food resources of varying seasonal availability, hence, opportunistic.

Present-day instances of hominoid direct sympatry usually occur between more folivorous vs more frugivorous taxa (gibbons and siamangs in Asia [49] and chimpanzees and gorillas in Africa [46]). In both extant cases the more folivorous species is twice as large as the more frugivorous one. At Hammerschmiede the sympatric hominids differ in size to a comparable degree without evidence of a strong frugivore-folivore dichotomy, but instead a divergence of dietary preferences that may have been more on the soft fruit to hard/tough object spectrum. This may be more analogous with orangutan/gibbon sympatry in Borneo and Sumatra [50]. The smaller taxon (*Hylobates*) with a preference for soft fruits feeds higher in the canopy than the much larger taxon (*Pongo*), which, with the differences in diet would have aided in avoiding competition [50]. This is consistent with our observation of a possible preference for higher canopy/terminal branch foraging in *Buronius*. Hypotheses of feeding ecology in *Buronius* will need to be tested with more specimens. More research is also needed both in reconstructing vegetation and fruit availability at Hammerschmiede. This is all the more necessary as the determining factors for primate species richness are the primary productivity [51], the forest structure [52] and its structural diversity [50].

## Conclusion

Two teeth and a patella from the HAM 5 level at Hammerschmiede are pliopithecoid in size but easily distinguished from that taxon in morphology. The unworn M^2^ crown is unlike pliopithecoids and *Pliobates*, having a reduced, smooth cingulum and buccal style and being narrower relative to length. Cristae are less strongly developed, cusps less compressed and basins broader and shallower. However, the cingulum, style and other occlusal details distinguish it from *Danuvius* and all other European hominines (or hominids, according to some [32]). The P_4_ fragment also lacks the typical pliopithecoid cristodonty and deep basins and is aligned more closely with hominids. Similarly, the patella differs from pliopithecoids and non-hominids by its roundish overall morphology. A pointed distal patellar apex and an asymmetric, saddle-shaped condylar surface is possibly suggestive of climbing locomotion with more abducted hindlimb postures. The morphology and size of this sample requires recognition of a new genus from the stratigraphic level at Hammerschmiede in which the *Danuvius* hypodigm was recovered. Dental and patellar morphology differ between *Buronius* and *Danuvius*, suggestive of differences in feeding ecology. The *Buronius* M^2^ is suggestive of a soft diet that may have also included a significant fibrous component, while in *Danuvius* the dentition is more consistent with a preference or ability to exploit hard/tough objects. Differences in patella morphology suggest more climbing with abducted hindlimb postures in *Buronius*, as opposed to the probably more cautious extended limb clambering of *Danuvius*. *Buronius* may have typically foraged higher in the canopy than *Danuvius*. With a body size of about 10 kg *Buronius* is the smallest crown-hominid known so far.

Two hominid genera at Hammerschmiede is unique among European middle and late Miocene localities (Paşalar in Anatolia also has two hominid genera, but not in direct sympatry [53]), but more like taxon diversity in African early and middle Miocene hominoid localities. A re-examination of morphological variation at other rich European Miocene localities may reveal heretofore unrecognized diversity in these samples as well (e.g. [2, 54]).

### Fossil repository

All described Hammerschmiede fossils are stored in the palaeontological collection of the University of Tübingen (acronym GPIT), a research infrastructure of the Senckenberg Institute for Human Evolution and Palaeoenvironment (SHEP) Tübingen.

## Supporting information

S1 Fig Measurements used to calculate and display the relative occlusal polygon area (ROPA), and protocone, and paracone angles. (PDF)

S2 Fig Anterior and posterior views of left patella of living and fossil catarrhines. A–*Nasalis larvatus* (ZSM 1907/3048), B–*Papio hamadryas* (RMCA A3.40.M.14), C–*Symphalangus syndactylus* (ZSM 1905/60), D–*Pongo pygmaeus* (ZSM 1909/801), E–*Pan paniscus* (RMCA 15293), F–*Ekembo heseloni* (KPS PT4), G–*Ekembo nyanzae* (KNM-RU 18384), H–*Buronius manfredschmidi* (GPIT/MA/10007), I–*Danuvius guggenmosi* (GPIT/MA/10000-12). All the patellas are from left side, except the anterior view in D, C and E. Anterior view is left, posterior view is right (except the anterior view only in D). Scale bar is 10 mm.
(PDF)

S3 Fig Occlusal views of upper molars of late Middle and late Miocene European hominines (or stem hominids). A: GPIT/MA/13005 (*Buronius*); B: GPIT/MA/10002-07 (*Danuvius*); C: Alsótelekes (*Rudapithecus*) M1-M2; D: GPIT/MA/2122 (Melchingen dryopithecin indet.); E: IPS 35026 (*Dryopithecus*); F: IPS 21350 (*Pierolapithecus*); G: IPS 43000 (*Anoiapithecus*), reversed; H: IPS 1815 (*Hispanopithecus*), modified from Alba et al. (2012). Note the large difference in size between *Buronius* and all other dryopithcins. The *Buronius* specimen has tall, pointed cusps with sharp principal crista and few assessory cristae (all other taxa have secondary cristae in the trigon, usually directed between the paracone and the lingual third of the crista obliqua), a lingually concave, notched, sharp postprotocone crista, a low and deeply notched hypocone-metacone crista, a rounded, shallow cingulum remnant, a shallow buccal shelf (style), no mesial fovea, and a strongly lingually positioned hypocone. Other differences include a shorter talon, lacking the distolingual expansion of *Danuvius*, *Dryopithecus*, *Pierolapithecus*, *Anoiapithecus* and *Hispanopithecus*; more obliquely oriented, continuous postparacone-prematacone cristae compared with all taxa except *Danuvius* (in *Danuvius* the cristae are separated by a fissure); continuous postprotocone-prehypocone cristae in contrast to the deep notch separating the crista in all taxa except the tooth from Melchingen.
(PDF)

S4 Fig Comparison of *Buronius manfredschmidi* with associated upper first and second molars of dryopithecins. All teeth from the left side except *Anoiapithecus*, which is photographically reversed. All specimens scaled to the same M2 size (scales = 10 mm). *Danuvius guggenmosi* (GPIT/MA/10000-01), *Rudapithecus hungaricus* (Alsótelekes), *Dryopithecus fontani* (IPS 35026), *Pierolapithecus catalaunicus* (IPS 21350), *Anoiapithecus brevirostris* (IPS 43000), *Hispanopithecus laietanus* (IPS 1798).
(PDF)

S5 Fig Metrical comparison of *Buronius manfredschmidi* to other dryopithecins. A–M1 Size: M1 mesiodistal length times buccolingual breadth (MD)*(BL). *Buronius* is much smaller than any known dryopithecin M1. B–M2 size: M2 mesiodistal length times buccolingual breadth (MD)*(BL). *Buronius* is much smaller than any known dryopithecin M2. C–M1 length/breadth (MD/BL) ratios in *Buronius* and dryopithecins. The *Buronius* M2 is relatively broad compared with *Anoiapithecus* and *Dryopithecus*, and within the 25–75 confidence intervals in other dryopithecins. D–M2 length/breadth (MD/BL) ratios in *Buronius* and dryopithecins. The *Buronius* M2 is relatively broad compared with *Danuvius* and *Dryopithecus*, and within the 25–75 confidence intervals in other dryopithecins.
(PDF)

S6 Fig Enamel thickness on unworn upper molars (A, B) compared to worn deciduous molar (C). Distal virtual section through the tips of metacone and hypocone for the left M2 of *Buronius manfredschmidi* (A, GPIT/MA/13005), the left M2 of *Danuvius guggenmosi* (B, GPIT-MA-10002-07), the right DP4 (reversed) of *Danuvius guggenmosi* (C, GPIT-MA-10002-04) and the left M2 of *Rudapithecus* (RUD 200; modified from Smith et al., 2019). Note the conservative reconstruction of worn enamel on the tips of metacone and hypocone in the deciduous molar (B). Scale bar is 2 mm.
(PDF)

S7 Fig Deciduous and permanent upper molar comparisons. Comparisons between the dP4 and M2 of selected catarrhines. Top row, left to right: A—*Buronius* (M2), B—*Danuvius* (dP4, reversed), C—*Danuvius* (M2), D—*Anapithecus* (M2), E—*Rudapithecus* (dP4, RUD 124). Box: dP4 of *Griphopithecus* (four teeth modified from Mortzou and Andrews (2008). Two teeth in the lower right corner are casts of *Griphopithecus* from Pasalar (left) and from Devinska Nova Nes (Slovakia) (right). Note that the dP4s are nearly always worn. The metacones are displaced lingually, contributing to a tapered crown distobuccally. The crowns are always flared, especially lingually. Trigons are usually short and crest poorly developed.
(PDF)

S8 Fig Lingual views of fossil hominoid dP4 compared with the M2 of *Buronius*. Upper left: *Danuvius* dP4 (GPIT-MA-10002-04); Upper right, *Buronius* M2 (GPIT/MA/13005); Lower left, *Griphopithecus* dP4 from Devinska Nova Ves; Middle: *Griphopithecus* dP4 from Pasalar; Lower right, *Rudapithecus* dP4. Note the much lower crowns of the dP4s.
(TIF)

S9 Fig Ratios of buccolingual breadth to mesiodistal length in selected hominoids. The dP4 tends to be broader relative to length but there is much variation and overlap with the permanent molars. *Pan paniscus* has relatively broad upper molars, like *Buronius*.
(TIF)

S10 Fig Lateral enamel thickness diphyodontic index (Zanolli et al. (2017). This is a measure of the ratio of enamel thickness between the lower dp4 and lower m1. Since enamel thickness does not vary much between M1 and M2 and since we are comparing the dP4 and upper M2 of *Buronius* and *Danuvius* this comparison is relevant. There is considerable variability in this ratio within the samples included in Zanolli et al. (2017). The ratio resulting from a theoretical pairing of the *Buronius* M2 and the *Danuvius* dP4 (B/D) in this plot) falls outside the range of variation of all samples included here, indicating that the difference in lateral enamel thickness is too great between *Buronius* and *Danuvius* to be accommodated within a single genus. Note as well that some thickly enameled hominins (modern *Homo*, *Australopithecus*) can have values close to one. The ratio in *Danuvius* falls closest to the mean for modern *Homo*).
(TIF)

S11 Fig Comparisons of the development of the protoconule in *Danuvius* and *Buronius*. The protoconule in the dP4 of *Danuvius* is worn (note the large pit at its apex). See S1 File for description.
(TIF)

S12 Fig Oblique views from distally showing the difference in development of the crista obliqua (yellow arrow) in *Danuvius* and *Buronius*. See S1 File for description.
(TIF)

S13 Fig The mesial fovea in relation to the protoconule. The mesial fovea does not reach the protoconule in *Danuvius* while it does in *Buronius*, as its mesial and distal borders converge lingually on to the tip of the protoconule. Despite damage, there is a distinct ridge mesiobuccal to the protoconule on the deciduous tooth corresponding to a similar ridge on the permanent tooth of *Danuvius*. See S1 File for description.
(TIF)

S14 Fig Enamel-dentine junction of the *Buronius* M2 (A, GPIT/MA/13005), compared to the dP4 (B, GPIT/MA/10002-04) and M2 (C, GPIT/MA/10002-07) of *Danuvius guggenmosi*. Not to scale. Images aligned along dentine horns of protocone and metacone. See S1 File for description.
(TIF)

S1 Table Summary of comparisons among the permanent molars of *Danuvius* and *Buronius* and the dP4 of *Danuvius*. MD–mesio-distal.
(DOCX)

S1 File EDJ of *Buronius* and *Danuvius*. (DOCX)

S2 File Description and comparison of the left upper M2 from *Danuvius guggenmosi* (GPIT-MA-10002-7). (DOCX)

S3 File PCA tables for Fig 9C. (DOCX)

S4 File (XLSX)
