# Supplementary figures and images for: Buronius manfredschmidi—A new small hominid from the early late Miocene of Hammerschmiede (Bavaria, Germany)
Source: PLoS One. 2024 Jun 7;19(6):e0301002. doi: 10.1371/journal.pone.0301002 (PMC11161025; doi:10.1371/journal.pone.0301002)

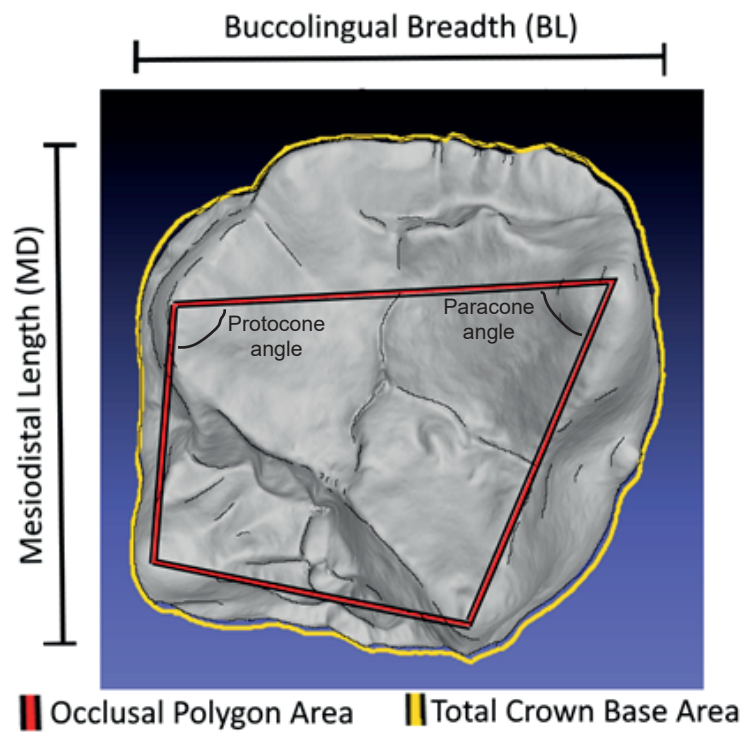

Supplement: S1 Fig — (PDF) [file pone.0301002.s001.pdf]

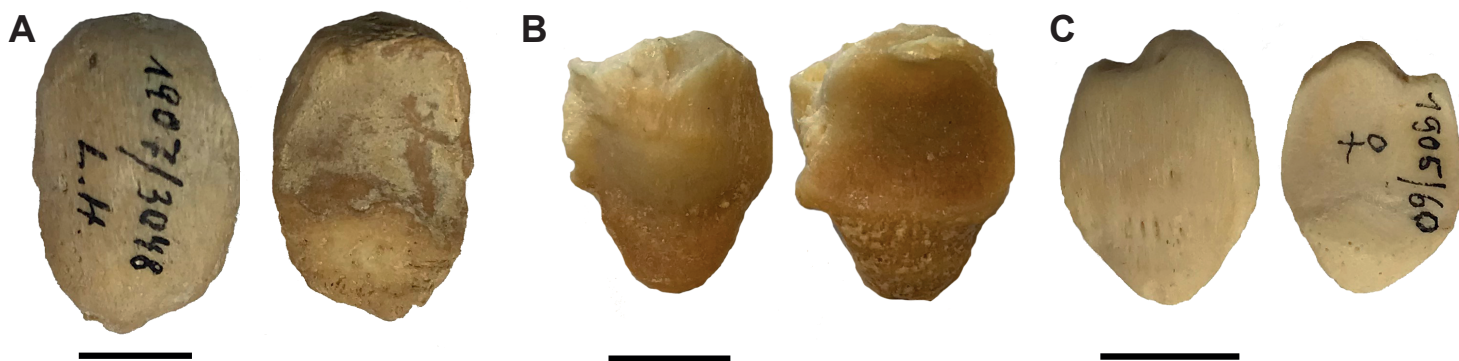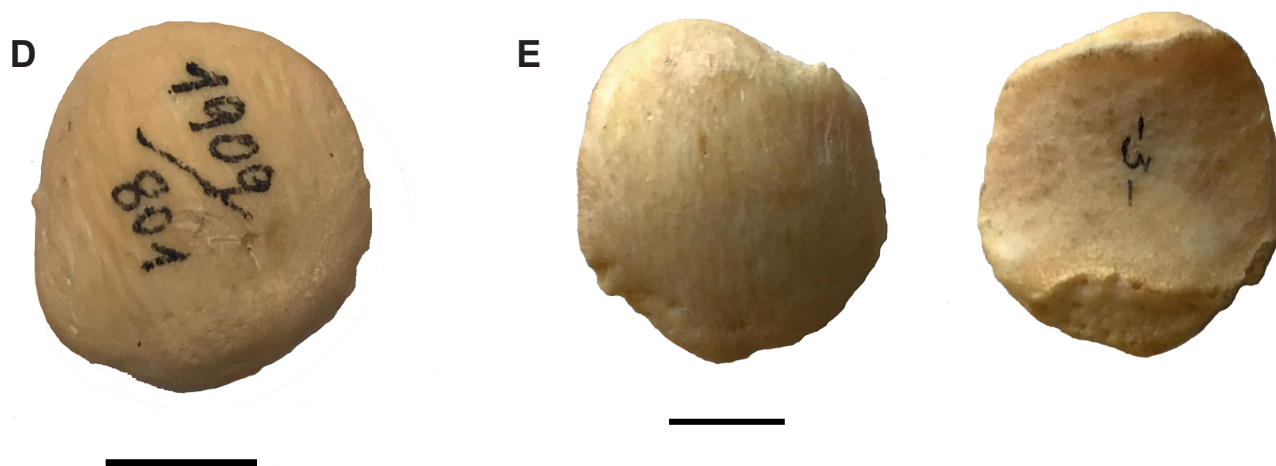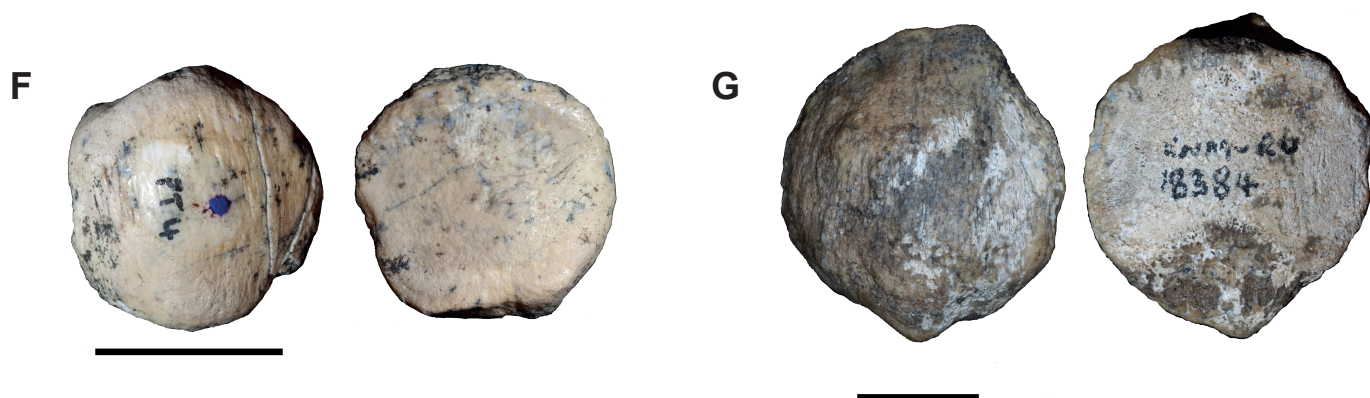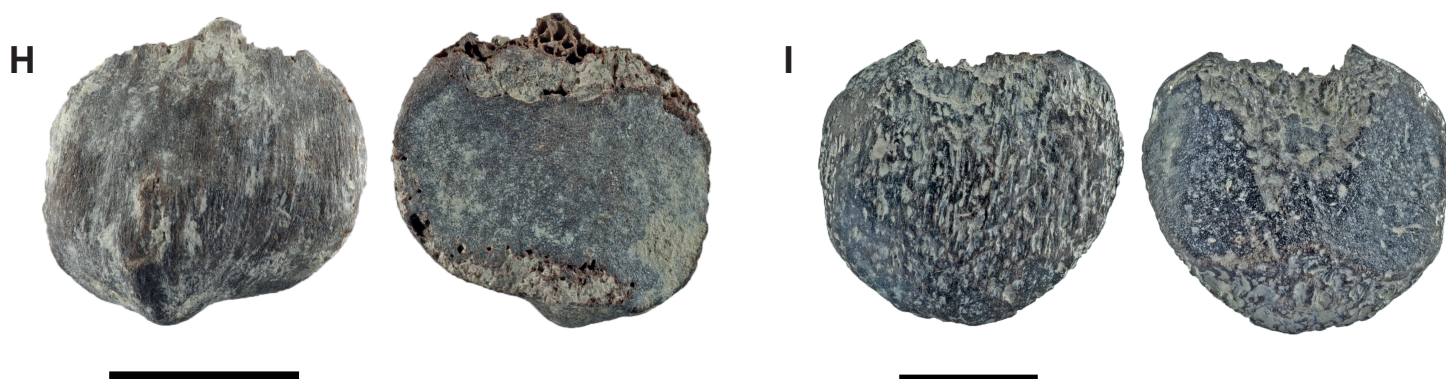

Supplement: S2 Fig — A–Nasalis larvatus (ZSM 1907/3048), B–Papio hamadryas (RMCA A3.40.M.14), C–Symphalangus syndactylus (ZSM 1905/60), D–Pongo pygmaeus (ZSM 1909/801), E–Pan paniscus (RMCA 15293), F–Ekembo heseloni (KPS PT4), G–Ekembo nyanzae (KNM-RU 18384), H–Buronius manfredschmidi (GPIT/MA/10007), I–Danuvius guggenmosi (GPIT/MA/10000-12). All the patellas are from left side, except the anterior view in D, C and E. Anterior view is left, posterior view is right (except the anterior view only in D). Scale bar is 10 mm. (PDF) [file pone.0301002.s002.pdf]

A

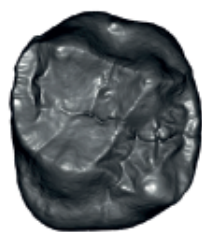

B

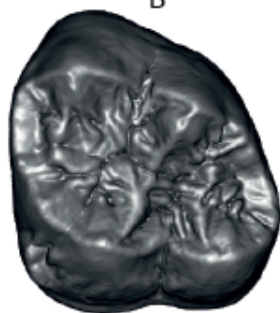

C

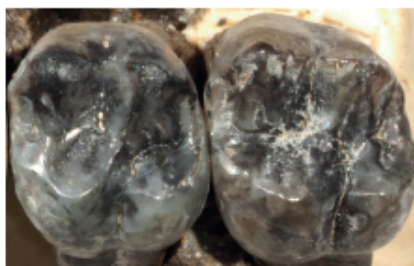

D

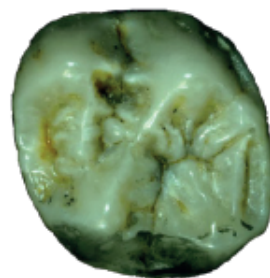

5mm

E

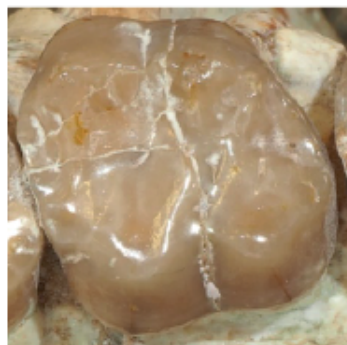

F

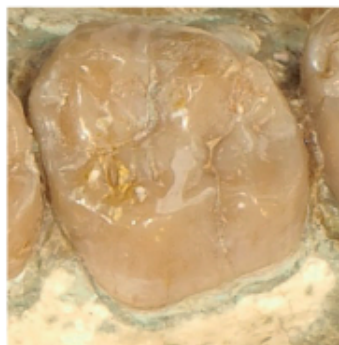

G

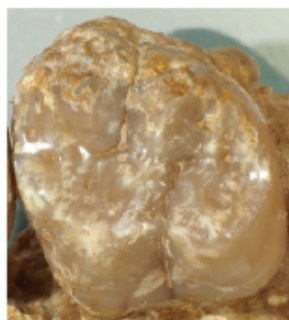

H

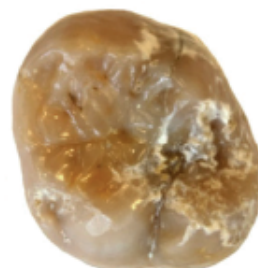

Supplement: S3 Fig — A: GPIT/MA/13005 (Buronius); B: GPIT/MA/10002-07 (Danuvius); C: Alsótelekes (Rudapithecus) M1-M2; D: GPIT/MA/2122 (Melchingen dryopithecin indet.); E: IPS 35026 (Dryopithecus); F: IPS 21350 (Pierolapithecus); G: IPS 43000 (Anoiapithecus), reversed; H: IPS 1815 (Hispanopithecus), modified from Alba et al. (2012). Note the large difference in size between Buronius and all other dryopithcins. The Buronius specimen has tall, pointed cusps with sharp principal crista and few assessory cristae (all other taxa have secondary cristae in the trigon, usually directed between the paracone and the lingual third of the crista obliqua), a lingually concave, notched, sharp postprotocone crista, a low and deeply notched hypocone-metacone crista, a rounded, shallow cingulum remnant, a shallow buccal shelf (style), no mesial fovea, and a strongly lingually positioned hypocone. Other differences include a shorter talon, lacking the distolingual expansion of Danuvius, Dryopithecus, Pierolapithecus, Anoiapithecus and Hispanopithecus; more obliquely oriented, continuous postparacone-prematacone cristae compared with all taxa except Danuvius (in Danuvius the cristae are separated by a fissure); continuous postprotocone-prehypocone cristae in contrast to the deep notch separating the crista in all taxa except the tooth from Melchingen. (PDF) [file pone.0301002.s003.pdf]

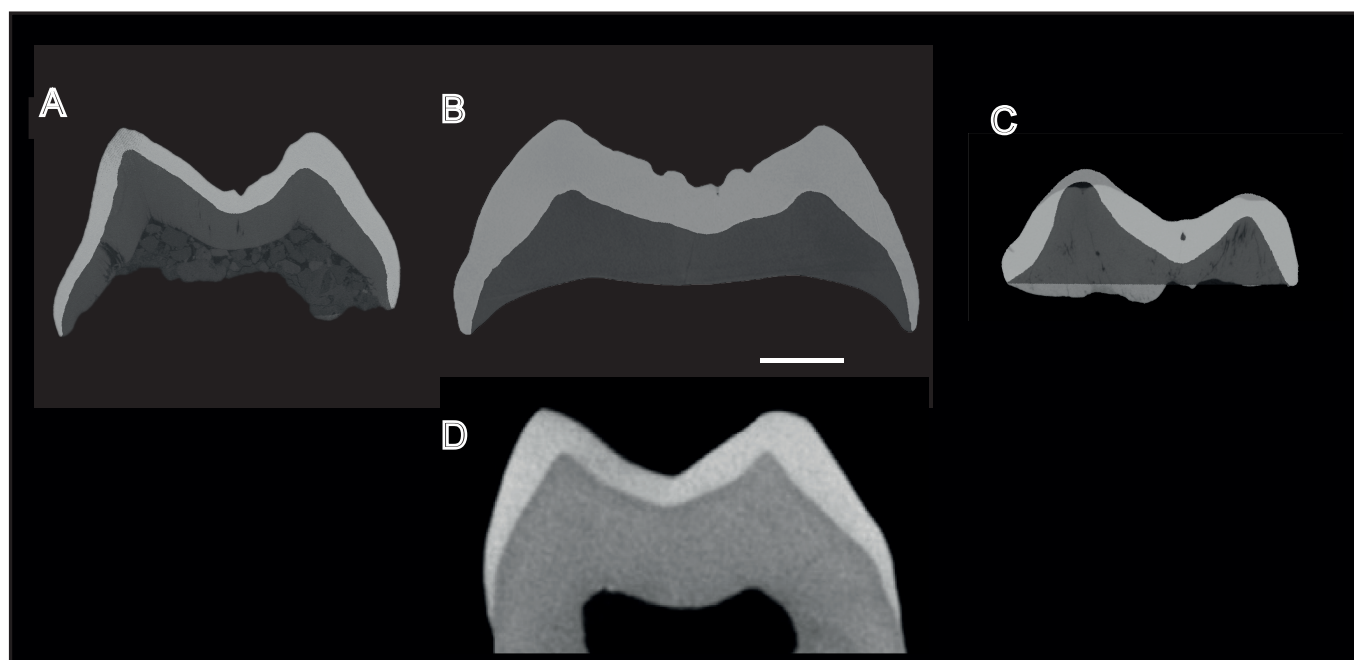

Supplement: S6 Fig — Enamel thickness on unworn upper molars (A, B) compared to worn deciduous molar (C). Distal virtual section through the tips of metacone and hypocone for the left M2 of Buronius manfredschmidi (A, GPIT/MA/13005), the left M2 of Danuvius guggenmosi (B, GPIT-MA-10002-07), the right DP4 (reversed) of Danuvius guggenmosi (C, GPIT-MA-10002-04) and the left M2 of Rudapithecus (RUD 200; modified from Smith et al., 2019). Note the conservative reconstruction of worn enamel on the tips of metacone and hypocone in the deciduous molar (B). Scale bar is 2 mm. (PDF) [file pone.0301002.s006.pdf]

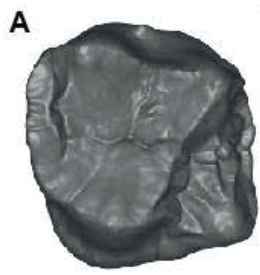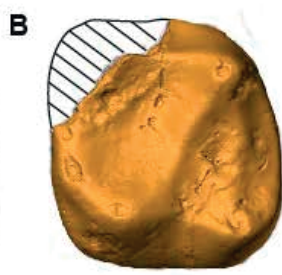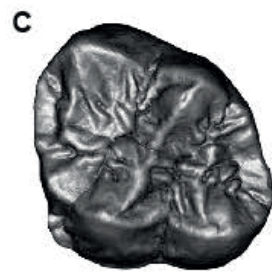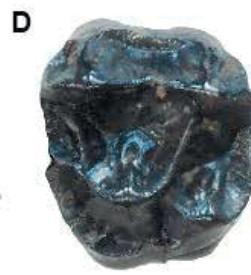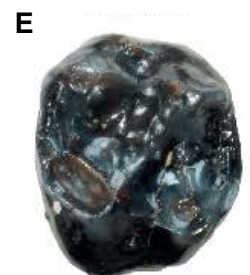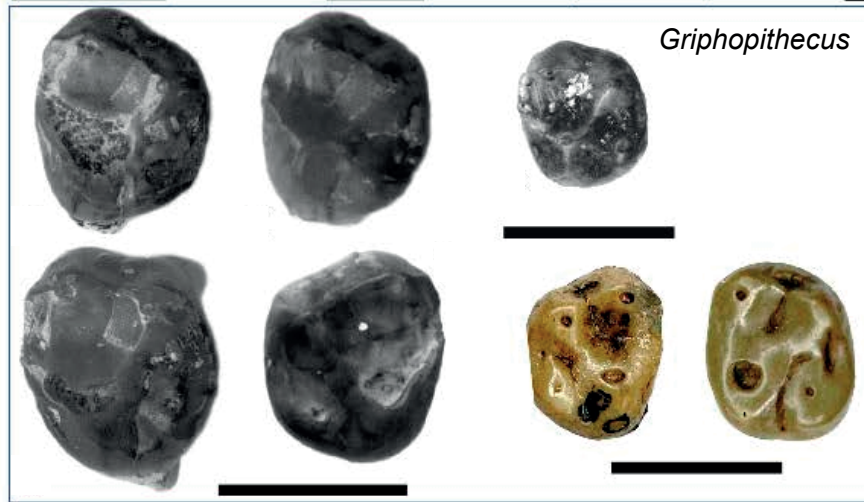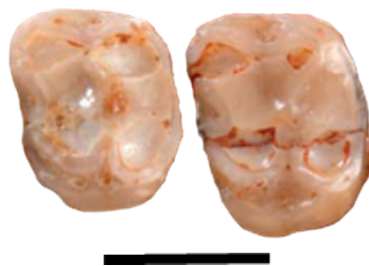

Supplement: S7 Fig — Comparisons between the dP4 and M2 of selected catarrhines. Top row, left to right: A—Buronius (M2), B—Danuvius (dP4, reversed), C—Danuvius (M2), D—Anapithecus (M2), E—Rudapithecus (dP4, RUD 124). Box: dP4 of Griphopithecus (four teeth modified from Mortzou and Andrews (2008). Two teeth in the lower right corner are casts of Griphopithecus from Pasalar (left) and from Devinska Nova Nes (Slovakia) (right). Note that the dP4s are nearly always worn. The metacones are displaced lingually, contributing to a tapered crown distobuccally. The crowns are always flared, especially lingually. Trigons are usually short and crest poorly developed. (PDF) [file pone.0301002.s007.pdf]

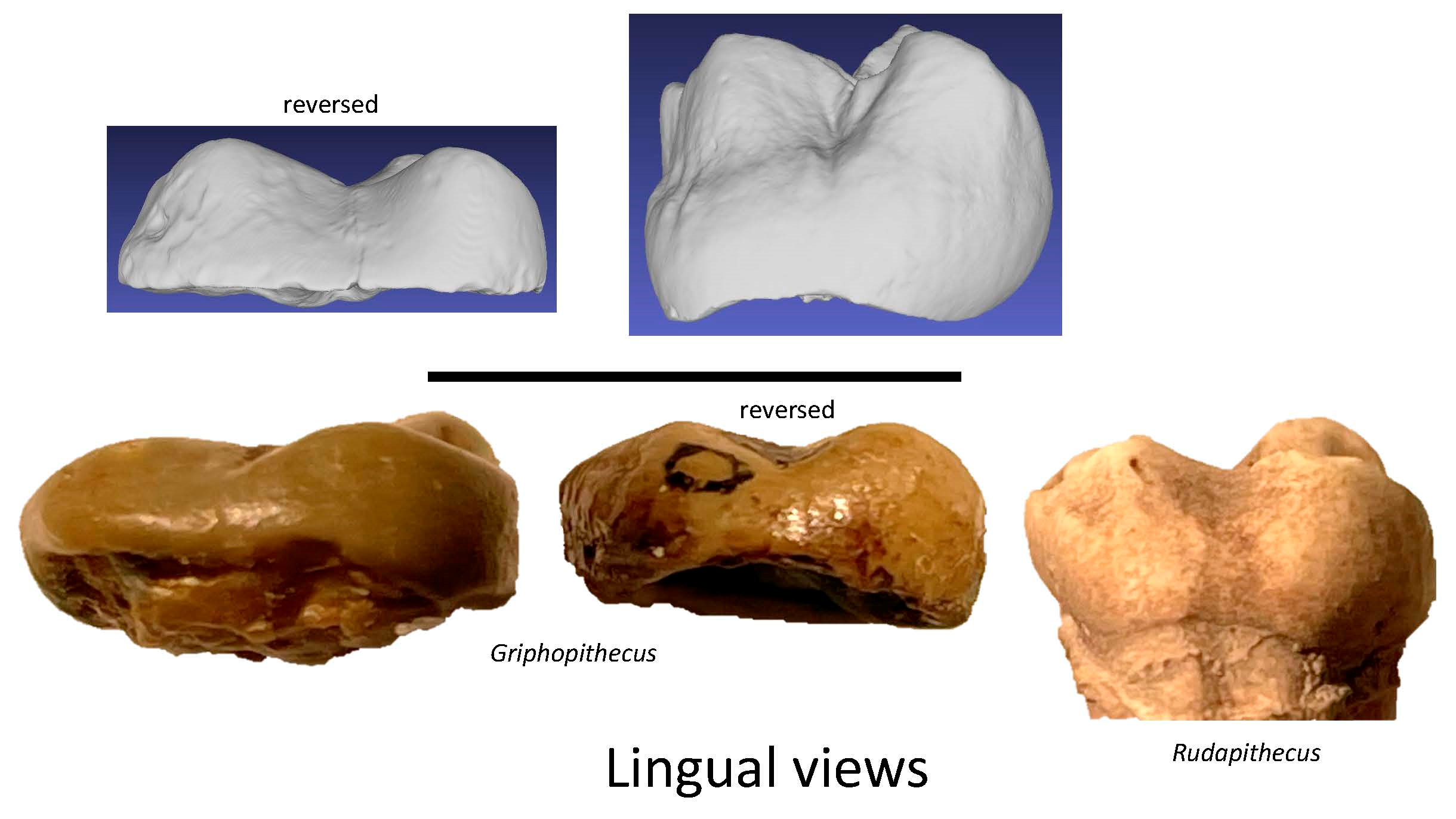

Supplement: S8 Fig — Upper left: Danuvius dP4 (GPIT-MA-10002-04); Upper right, Buronius M2 (GPIT/MA/13005); Lower left, Griphopithecus dP4 from Devinska Nova Ves; Middle: Griphopithecus dP4 from Pasalar; Lower right, Rudapithecus dP4. Note the much lower crowns of the dP4s. (TIF) [file pone.0301002.s008.tif]

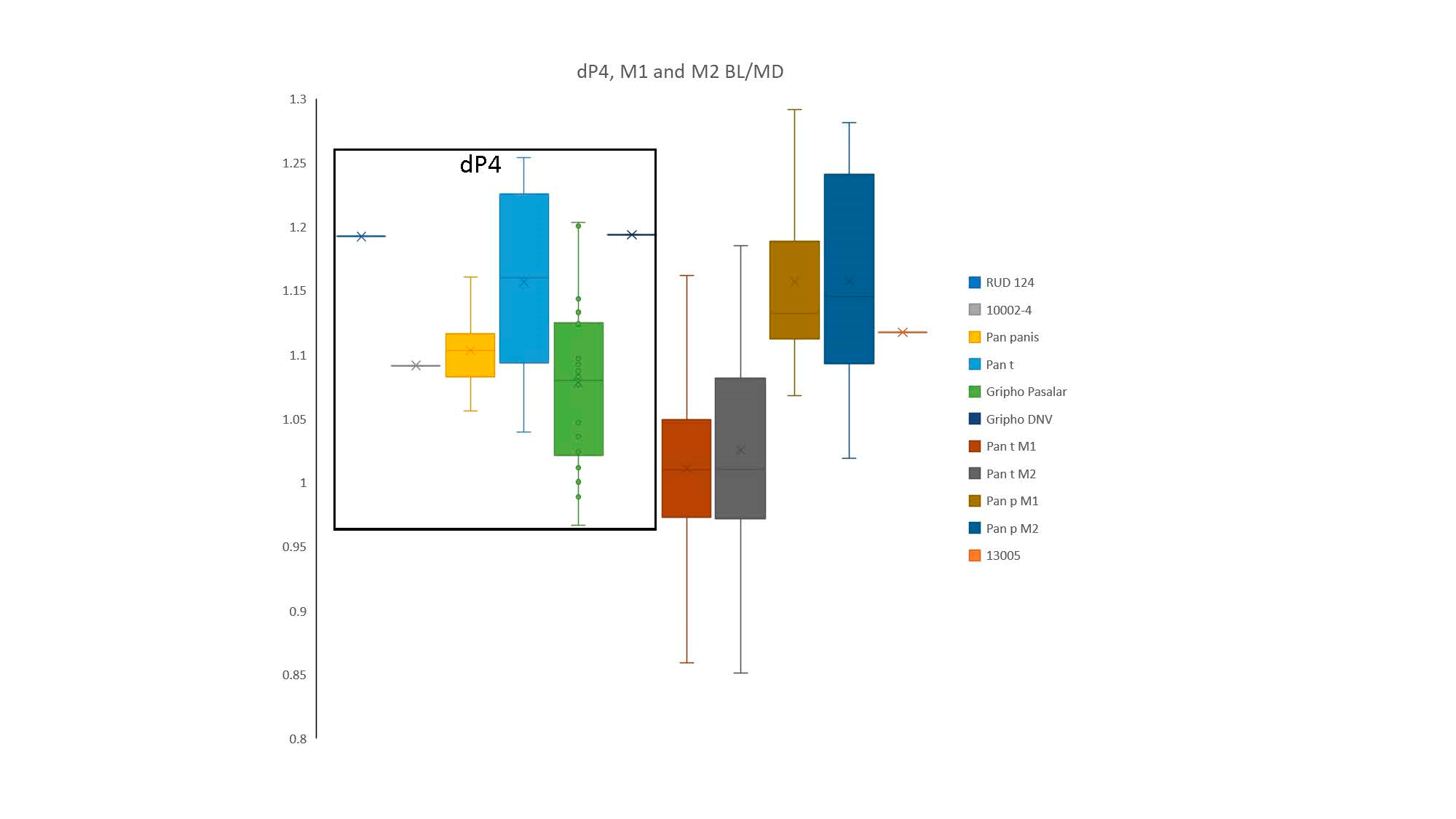

Supplement: S9 Fig — The dP4 tends to be broader relative to length but there is much variation and overlap with the permanent molars. Pan paniscus has relatively broad upper molars, like Buronius. (TIF) [file pone.0301002.s009.tif]

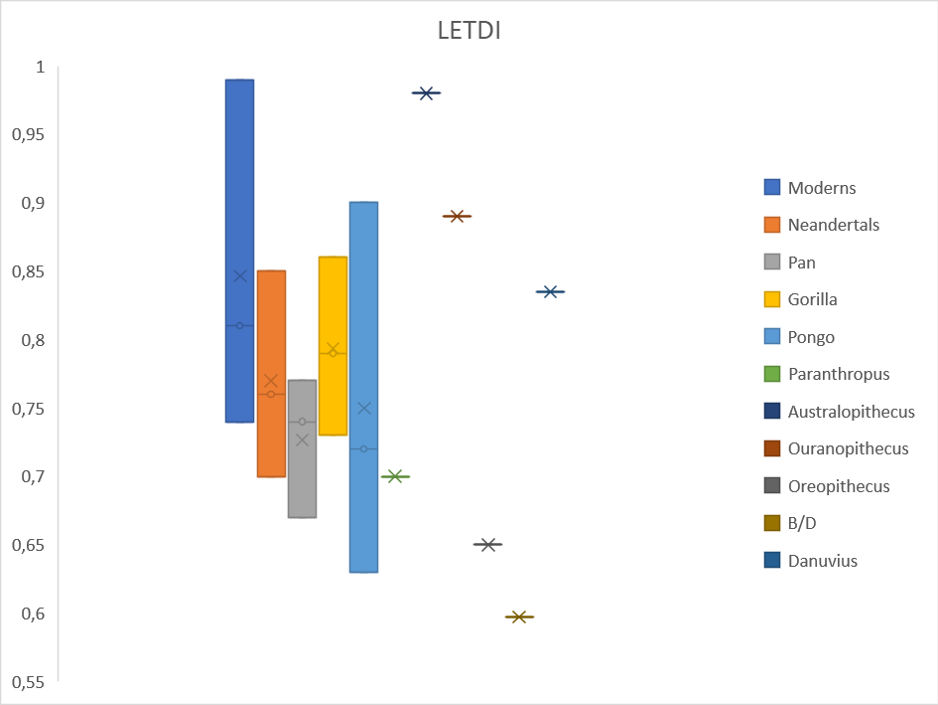

Supplement: S10 Fig — This is a measure of the ratio of enamel thickness between the lower dp4 and lower m1. Since enamel thickness does not vary much between M1 and M2 and since we are comparing the dP4 and upper M2 of Buronius and Danuvius this comparison is relevant. There is considerable variability in this ratio within the samples included in Zanolli et al. (2017). The ratio resulting from a theoretical pairing of the Buronius M2 and the Danuvius dP4 (B/D) in this plot) falls outside the range of variation of all samples included here, indicating that the difference in lateral enamel thickness is too great between Buronius and Danuvius to be accommodated within a single genus. Note as well that some thickly enameled hominins (modern Homo, Australopithecus) can have values close to one. The ratio in Danuvius falls closest to the mean for modern Homo). (TIF) [file pone.0301002.s010.tif]

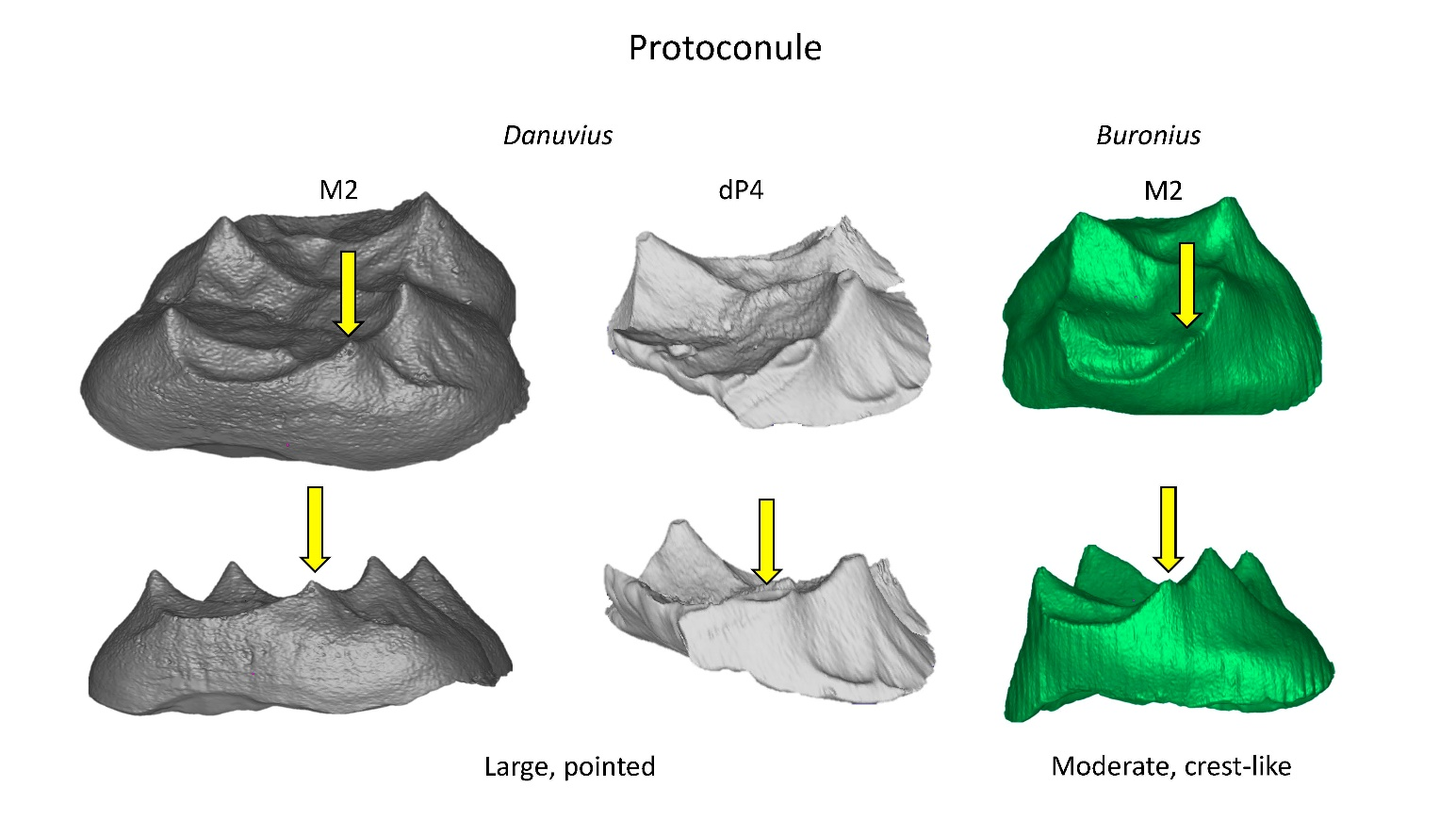

Supplement: S11 Fig — The protoconule in the dP4 of Danuvius is worn (note the large pit at its apex). See S1 File for description. (TIF) [file pone.0301002.s011.tif]

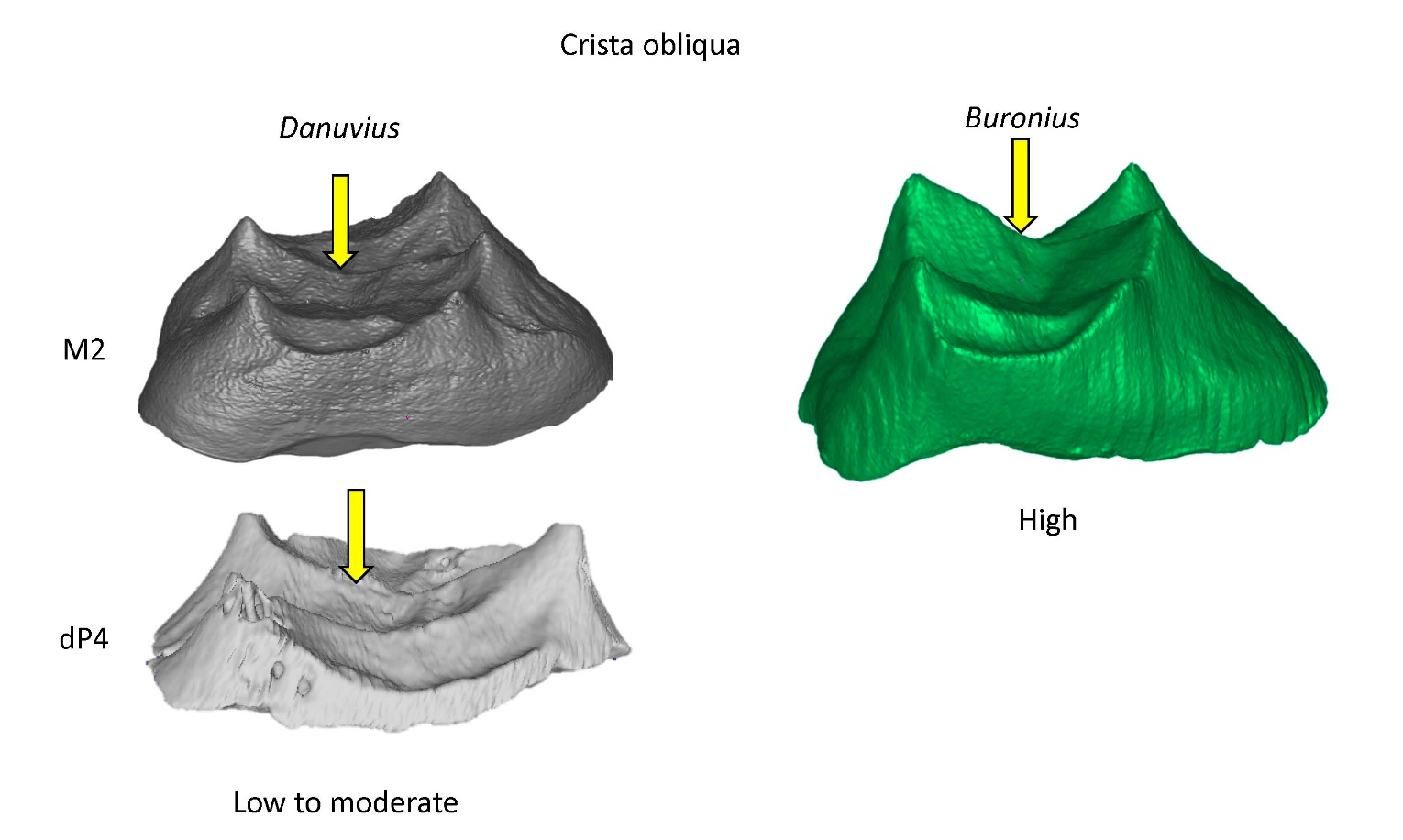

Supplement: S12 Fig — See S1 File for description. (TIF) [file pone.0301002.s012.tif]

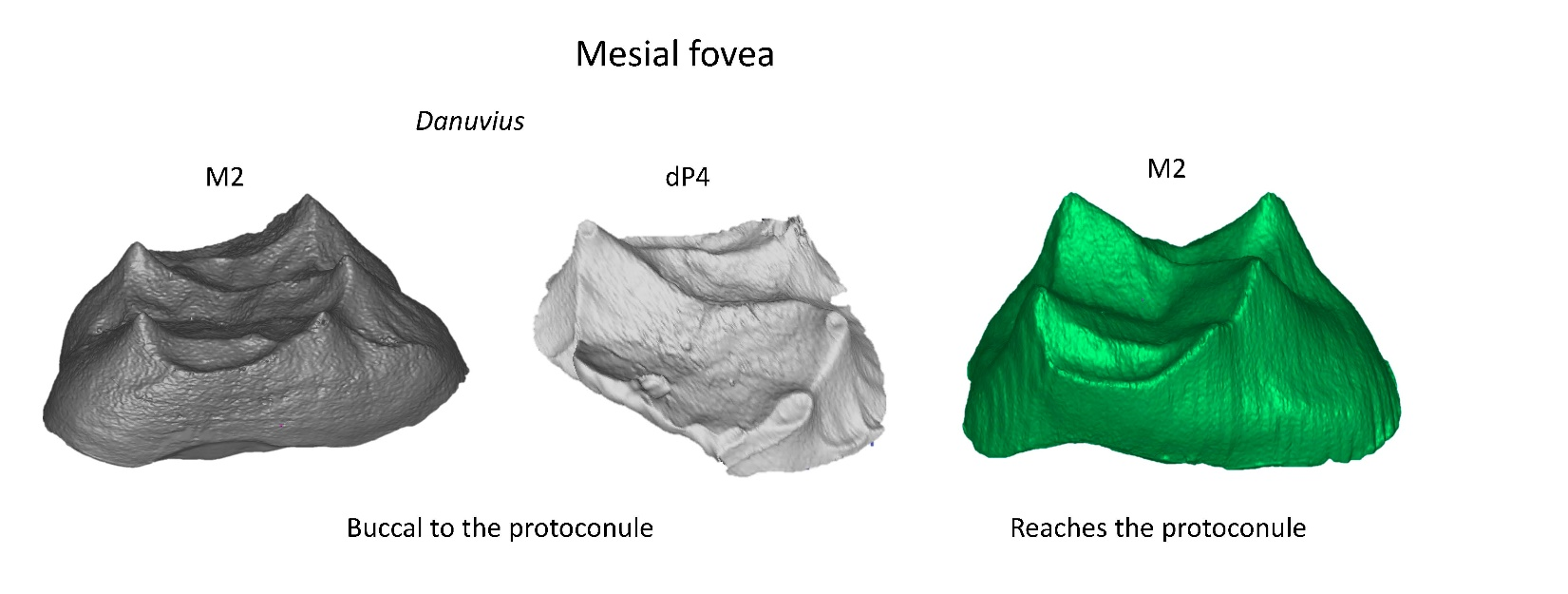

Supplement: S13 Fig — The mesial fovea does not reach the protoconule in Danuvius while it does in Buronius, as its mesial and distal borders converge lingually on to the tip of the protoconule. Despite damage, there is a distinct ridge mesiobuccal to the protoconule on the deciduous tooth corresponding to a similar ridge on the permanent tooth of Danuvius. See S1 File for description. (TIF) [file pone.0301002.s013.tif]

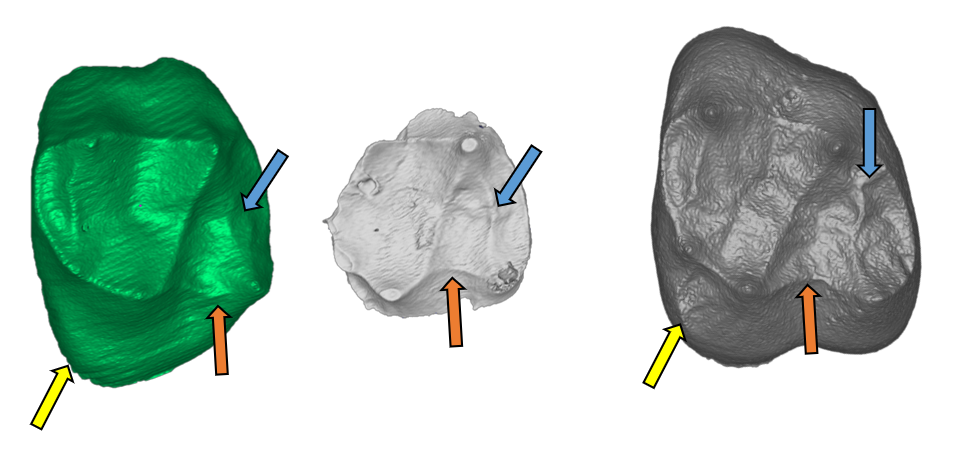

Supplement: S14 Fig — Not to scale. Images aligned along dentine horns of protocone and metacone. See S1 File for description. (TIF) [file pone.0301002.s014.tif]
