# Supplementary material for: Buronius manfredschmidi—A new small hominid from the early late Miocene of Hammerschmiede (Bavaria, Germany)
Source: PLoS One. 2024 Jun 7;19(6):e0301002. doi: 10.1371/journal.pone.0301002 (PMC11161025; doi:10.1371/journal.pone.0301002)
Supplement: S4 Fig — All teeth from the left side except Anoiapithecus, which is photographically reversed. All specimens scaled to the same M2 size (scales = 10 mm). Danuvius guggenmosi (GPIT/MA/10000-01), Rudapithecus hungaricus (Alsótelekes), Dryopithecus fontani (IPS 35026), Pierolapithecus catalaunicus (IPS 21350), Anoiapithecus brevirostris (IPS 43000), Hispanopithecus laietanus (IPS 1798). (PDF) [file pone.0301002.s004.pdf]

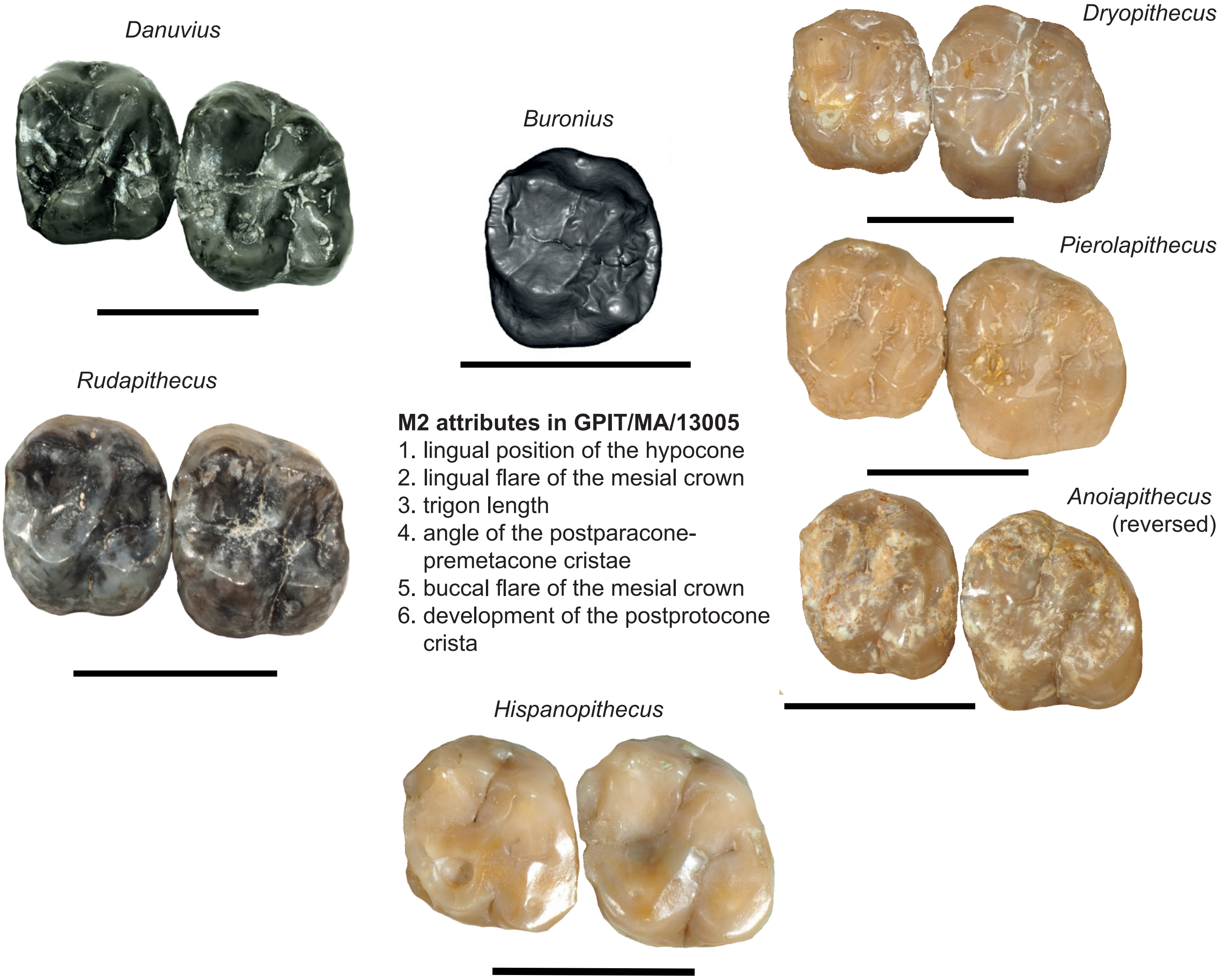

Danuvius
Buronius
Dryopithecus
Pierolapithecus
Rudapithecus
M2 attributes in GPIT/MA/13005
1. lingual position of the hypocone
2. lingual flare of the mesial crown
3. trigon length
4. angle of the postparacone-premetacone cristae
5. buccal flare of the mesial crown
6. development of the postprotocone crista
Anoiapithecus (reversed)
Hispanopithecus
