# Supplementary material for: Buronius manfredschmidi—A new small hominid from the early late Miocene of Hammerschmiede (Bavaria, Germany)
Source: PLoS One. 2024 Jun 7;19(6):e0301002. doi: 10.1371/journal.pone.0301002 (PMC11161025; doi:10.1371/journal.pone.0301002)
Supplement: S5 Fig — A–M1 Size: M1 mesiodistal length times buccolingual breadth (MD)*(BL). Buronius is much smaller than any known dryopithecin M1. B–M2 size: M2 mesiodistal length times buccolingual breadth (MD)*(BL). Buronius is much smaller than any known dryopithecin M2. C–M1 length/breadth (MD/BL) ratios in Buronius and dryopithecins. The Buronius M2 is relatively broad compared with Anoiapithecus and Dryopithecus, and within the 25–75 confidence intervals in other dryopithecins. D–M2 length/breadth (MD/BL) ratios in Buronius and dryopithecins. The Buronius M2 is relatively broad compared with Danuvius and Dryopithecus, and within the 25–75 confidence intervals in other dryopithecins. (PDF) [file pone.0301002.s005.pdf]

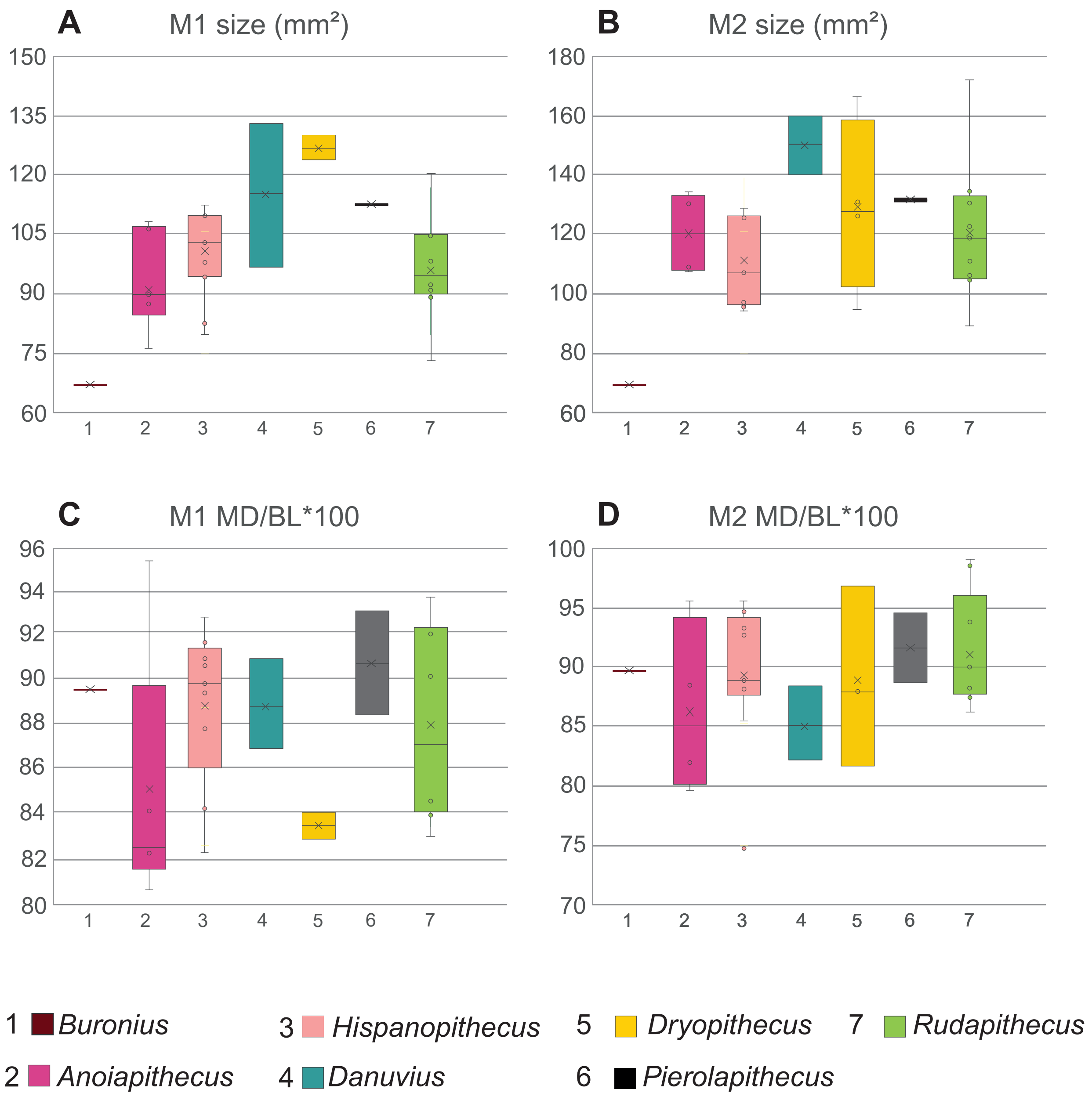
A
M1 size (mm²)
150
135
120
105
90
75
60
1 2 3 4 5 6 7
B
M2 size (mm²)
180
160
140
120
100
80
60
1 2 3 4 5 6 7
C
M1 MD/BL*100
96
94
92
90
88
86
84
82
80
1 2 3 4 5 6 7
D
M2 MD/BL*100
100
95
90
85
80
75
70
1 2 3 4 5 6 7
1 Buronius
2 Anoiapithecus
3 Hispanopithecus
4 Danuvius
5 Dryopithecus
6 Pierolapithecus
7 Rudapithecus
