# Supplementary material for: Buronius manfredschmidi—A new small hominid from the early late Miocene of Hammerschmiede (Bavaria, Germany)
Source: PLoS One. 2024 Jun 7;19(6):e0301002. doi: 10.1371/journal.pone.0301002 (PMC11161025; doi:10.1371/journal.pone.0301002)
Supplement: S1 Table — MD–mesio-distal. (DOCX) [file pone.0301002.s015.docx]

|  | ***Danuvius* dP4** | ***Danuvius* M2** | ***Buronius* M2** |
| --- | --- | --- | --- |
| **features** | **GPIT/MA/10002-04** | **GPIT/MA/10002-07** | **GPIT/MA/13005** |
| **dentine horns** | very peripheral and low | very peripheral and low | very peripheral and high |
| **mesial fovea** | lingually short, hypoparacrista terminates buccally to protoconule | deep and restricted, lingually shorter; hypoparacrista terminates in mesial marginal ridge buccally to protoconule | deep and restricted, linguallay expanded; hypoparacrista terminates in protoconule |
| **distal fovea** | present (hypocone-metacone crista) | present (hypocone-metacone crista) | absent |
| **trigon basin** |  | shallower | deeper |
| **talon basin** | MD longer | MD longer | MD shorter |
| **crista obliqua** | complete, low/moderately high | complete, low/moderately high | complete, high |
| **lingual cinculum** | absent | slightly developed | absent |
| **buccolingual waisting** | absent | marked | absent, very weak buccally |
| **protoconule** | large size, projecting cusp | large size, projecting cusp | moderate size, crest-like cusp |
