# Supplementary material for: Buronius manfredschmidi—A new small hominid from the early late Miocene of Hammerschmiede (Bavaria, Germany)
Source: PLoS One. 2024 Jun 7;19(6):e0301002. doi: 10.1371/journal.pone.0301002 (PMC11161025; doi:10.1371/journal.pone.0301002)
Supplement: S1 File — (DOCX) [file pone.0301002.s016.docx]

**EDJ of *Buronius* and *Danuvius***

The morphology of the EDJ distinguishes both the dP4 and the M2 of *Danuvius* from the M2 of *Buronius*. *Buronius* shows higher dentine horns and a smaller protoconule (yellow arrow) (grade 2 sensu Ortiz et al. 2019) (S11 Fig). The crista obliqua in *Danuvius* is lower and less trenchant than in *Buronius,* in which it is very prominent and sharp (S12 Fig). Its mesial fovea is more expanded lingually than in *Danuvius*, in which the hypoparacrista, which forms the distal margin of the mesial fovea, meets the mesial marginal ridge (the mesial margin of the fovea), buccal to the protoconule (S12 Fig). Furthermore, *Buronius* has a deeper trigon and mesiodistally shorter talon basin. It lacks a hypocone-metacone crista and hence a well delineated distal fovea (S13 Fig, blue arrows). *Danuvius* has a short lingual cingulum on the mesiolingual corner, as on the occlusal surface. This contrasts with *Buronius*, which lacks the ridge on the EDJ surface corresponding to the mild mesiolingual cingulum on the occlusal surface (S13 Fig, yellow arrow). Finally, in *Danuvius* the postprotocone crista and prehypocone crista are not aligned, but meet buccal to the cusp tips, forming a well-defined notch. In *Buronius* the corresponding crista is short and convex lingually (S13 Fig, orange arrow).

Importantly, the last deciduous molar of *Danuvius* agrees in EDJ morphology with the permanent molar in following features: low dentine horns, lingually short mesial fovea, well developed distal fovea, mesio-distal long talon basin, lower crista obliqua and a large protoconule (S1 Tab).
