# Supplementary material for: Buronius manfredschmidi—A new small hominid from the early late Miocene of Hammerschmiede (Bavaria, Germany)
Source: PLoS One. 2024 Jun 7;19(6):e0301002. doi: 10.1371/journal.pone.0301002 (PMC11161025; doi:10.1371/journal.pone.0301002)
Supplement: S2 File — (DOCX) [file pone.0301002.s017.docx]

**Description and comparison of the left upper M^2^ GPIT-MA-10002-7**

This specimen was found in summer 2019 just after submission of the manuscript Böhme et al. 2019, and belongs very probably to the juvenile paratype GPIT/MA/10002 of *Danuvius* *guggenmosi*. As it is figured in comparison with GPIT/MA/13005 in Fig. 5 of this publication it is described for the first time here.

**Description**. The tooth crown (MD 11.1 mm, BLm 12.7 mm, BLd 10.9 mm) was not fully erupted and consequently shows no wear and no mesial interstidal facet. The outline of the tooth is trapezoid, with a narrow and slightly tapering distal half. Cusps are strongly peripheralized, except the metacone. The protocone is the largest cusp, followed by the paracone and the hypocone. The metacone is lingually displaced and connected to the prae- and postmetacristae by a short transversal ridge. The postmetacrista runs towards a faint metaconule. Anteromesial to the protocone a large protoconule exists. On the mesiolingual tooth corner appears a well-developed, elongated tubercle of Carabelli. The mesial fovea is short and narrow and restricted to the buccal moiety of the mesial tooth border. The fovea is mesially restricted by a strong preparacrista and a weaker mesial branch of the hypoparacrista, which do not connect the protoconule. The short distal branch of the hypoparacrista ends in the trigone basin, together with the distal end of its mesial branch and the hypoprotoconulocrista. The crista transversa is well-developed. The floor of the talon basin (= distal fovea) is not divided by a crest, but filled with enamel crenulations and small tubercles. A weak lingual cingulum is present, which is hardly visible on the dentine surface.

**Comparison**. The GPIT-MA-10002-07 tooth morphology resembles the slightly worn M2 of the holotype individual (GPIT-MA-10000-1). It also compares well with late Miocene drypithecine genera such as *Rudapithecus* and *Hispanopithecus* and the isolated M2 from Hostalets (Alba et al. 2012, 2013) by well-peripheralized cusps. It differs from all Spanish hominids in the following features: the distal fovea is not divided on enamel surface by a hypocone-metacone crista (less developed in *Hispanopithecus*), the hypoparacrista is divided into two branches and do not connect to the protoconule, the possession of a Carabelli‘s tubercle (although a variable feature) and the separation of the metacone enamel cusp from its mesial and distal cristae. It more closely matches RUD 85, a large M^2^ from Rudabánya currently attributed to *Rudapithecus*. Both share a Carabelli’s tubercle, widely peripheralized cusps, an incompletely formed hypocone-metacone crista and a bifurcated hypoparacrista. However, RUD 85 is distinguished from other *Rudapithecus* and from *Danuvius* in having a crista obliqua that meets the postprotocone crista distal to the cusp apex, as in *Buronius*. *Anoiapithecus* differs in its more expanded protocone (para- and metacone highest cusps), *Pierolapithecus* differs by a less tapering M2 and more inflated crests, and *Dryopithecus* differs by a more expanded hypocone. The new *Danuvius* M^2^ strongly differs from *Griphopithecus*, which shows more centralized cusps, a narrow trigone basin, a stronger cingulum, a weak protoconule, an incipient mesial fovea and the division of the distal fovea by a strong hypohypocrista (= prehypocrista in Alba et al. 2013).

**Reference (for all Supporting Information)**

Alba, D.M. et al. 2013. New dental remains of Anoiapithecus and the first appearance datum of hominoids in the Iberian Peninsula. Journal of Human Evolution 65: 573-584.

distinguished from similarly sized pliopithecoids hominoids. It is small, below the range of variation of *Symphalangus*, in contrast to the M^1^. (fig. 2C.) GPIT-MA 12144 is labiolingually thick, falling closest to the thickest specimens of *Rudapithecus, Gorilla* and *Pan* (fig. 2D.)

The P^4^ fragment, GPIT-MA 13004 , is missing most of the lingual half of the crown. As with the other specimens, this P^4^  differs morphologically from pliopithecoids in crista, basin and cups morphology.

**Discussion: While clearly different from pliopithecoids, the small hominoids from HAM 5 are also distinguished from most Miocene apes. There is no close match with early and middle Miocene apes in quantitative morphology or in the morphology of the cusps, crista and basins (fig. 3.) The closest morphological match is with late Miocene European apes and extant hominoids (fig. 3). Like late Miocene hominines the cusps in GPIT-MA 13005 are widely spaced, the trigon is spacious and the crista obliqua is sharply defined.**

ADD QUANTITATIVE ANALYSIS FROM ANDREW.

**Results: The M^1^ (GPIT-MA 13005) is similar in size with large pliopithecoids and siamangs (fig. 2A.) It is unlikely to be a small *Danuvius* (fig. 2A, blue arrow.) It is narrower than pliopithecoids, within the range of variation of *Symphalangus* and the upper quartile of *Pan.* (fig. 2B.) GPIT-MA 13005 is quite different in occlusal morphology from all early and middle Miocene catarrhines and most closely resembles late Miocene apes. (fig. 3.) The M^1^ is unlike pliopithecoids and *Pliobates*, in having a reduced, smooth cingulum and buccal style and being narrower relative to length (fig. 1.) Crista are less strongly developed, cusps less compressed and basins broader and shallower. The cingulum and style distinguish it from *Danuvius* and all European hominines. The I^2^, which is missing its apex, is unlike pliopithecoids in being thickened at the cervix with a taller shoulder and a more convex lingual surface. The P^4^ fragment lacks the typical pliopithecoid cristodonty and deep basins. The I^2^, GPIT-MA 12144, is also morphologically and quantitatively**

distinguished from similarly sized pliopithecoids hominoids. It is small, below the range of variation of *Symphalangus*, in contrast to the M^1^. (fig. 2C.) GPIT-MA 12144 is labiolingually thick, falling closest to the thickest specimens of *Rudapithecus, Gorilla* and *Pan* (fig. 2D.)

The P^4^ fragment, GPIT-MA 13004 , is missing most of the lingual half of the crown. As with the other specimens, this P^4^  differs morphologically from pliopithecoids in crista, basin and cups morphology.

**Discussion: While clearly different from pliopithecoids, the small hominoids from HAM 5 are also distinguished from most Miocene apes. There is no close match with early and middle Miocene apes in quantitative morphology or in the morphology of the cusps, crista and basins (fig. 3.) The closest morphological match is with late Miocene European apes and extant hominoids (fig. 3). Like late Miocene hominines the cusps in GPIT-MA 13005 are widely spaced, the trigon is spacious and the crista obliqua is sharply defined.**

ADD QUANTITATIVE ANALYSIS FROM ANDREW.

**Results: The M^1^ (GPIT-MA 13005) is similar in size with large pliopithecoids and siamangs (fig. 2A.) It is unlikely to be a small *Danuvius* (fig. 2A, blue arrow.) It is narrower than pliopithecoids, within the range of variation of *Symphalangus* and the upper quartile of *Pan.* (fig. 2B.) GPIT-MA 13005 is quite different in occlusal morphology from all early and middle Miocene catarrhines and most closely resembles late Miocene apes. (fig. 3.) The M^1^ is unlike pliopithecoids and *Pliobates*, in having a reduced, smooth cingulum and buccal style and being narrower relative to length (fig. 1.) Crista are less strongly developed, cusps less compressed and basins broader and shallower. The cingulum and style distinguish it from *Danuvius* and all European hominines. The I^2^, which is missing its apex, is unlike pliopithecoids in being thickened at the cervix with a taller shoulder and a more convex lingual surface. The P^4^ fragment lacks the typical pliopithecoid cristodonty and deep basins. The I^2^, GPIT-MA 12144, is also morphologically and quantitatively**

Mortzou, Georgia, and Peter Andrews. “The Deciduous Dentition of Griphopithecus Alpani from Paşalar, Turkey.” *Journal of Human Evolution* 54, no. 4 (2008): 494–502. <https://doi.org/10.1016/j.jhevol.2007.08.001>.

Ortiz, A., Bailey, S.A., Delgado, M., Zanolli, C., Demeter, F., Bacon, A.-M., Nguyen, Thi M.H., Nguyen, Anh T., Zhang, Y., Harrison, T., Hublin, J.-J., Skinner, M.M. 2019. A distinguishing feature of Pongo upper molars and its implications for the taxonomic identification of isolated hominid teeth from the Pleistocene of Asia. American Journal of Physical Anthropology <https://doi.org/10.1002/ajpa.23928>

Zanolli, Clement, Priscilla Bayle, Luca Bondioli, M. Christopher Dean, Mona Le Luyer, Arnaud Mazurier, Wataru Morita, and Roberto Macchiarelli. “Is the Deciduous/Permanent Molar Enamel Thickness Ratio a Taxon-Specific Indicator in Extant and Extinct Hominids?” *Comptes Rendus. Palevol* 16, no. 5–6 (2017): 702–14. <https://doi.org/10.1016/j.crpv.2017.05.002>.
