# Supplementary material for: Buronius manfredschmidi—A new small hominid from the early late Miocene of Hammerschmiede (Bavaria, Germany)
Source: PLoS One. 2024 Jun 7;19(6):e0301002. doi: 10.1371/journal.pone.0301002 (PMC11161025; doi:10.1371/journal.pone.0301002)
Supplement: S3 File — (DOCX) [file pone.0301002.s018.docx]

**PCA tables for Fig. 9E**

PC Eigenvalue % variance

1 51.2738 54.705

2 23.411 24.978

3 16.2676 17.356

4 2.56529 2.7369

5 0.210572 0.22466

PC 1 PC 2 PC 3 PC 4 PC 5

MD 0.026374 0.026015 -0.019041 0.72501 -0.68748

BL 0.021883 0.014599 -0.056446 0.68526 0.72563

ROPA 0.17495 0.72451 0.6662 0.0081514 0.024272

Pr angle 0.96907 -0.010594 -0.24223 -0.045693 -0.0047025

Pa angle 0.17063 -0.68854 0.70282 0.051203 0.015024
